# Supplementary material for: The Evolution and Characterization of the RNA Interference Pathways in Lophotrochozoa
Source: Genome Biol Evol. 2024 May 7;16(5):evae098. doi: 10.1093/gbe/evae098 (PMC11114477; doi:10.1093/gbe/evae098)
Supplement: evae098_Supplementary_Data [file evae098_supplementary_data.zip › Supplementary_materials.zip!/selected_miRNAs/NoGe_selected_miRNAs/mirdeep_pdf_files/NMRB01001187.1_Notospermus_geniculatus_strain_Ushimado_scaffold1187,_whole_genome_shotgun_sequence_26006.pdf]

Provisional ID	:	NMRB01001187.1_Notospermus_geniculatus_strain_Ushimado_scaffold1187,_whole_genome_shotgun_sequence_26006
Score total	:	2238255.3
Score for star read(s)	:	3.9
Score for read counts	:	2238247.1
Score for mfe	:	1.2
Score for randfold	:	0
Score for cons. seed	:	3
Total read count	:	4390237
Mature read count	:	4388416
Loop read count	:	0
Star read count	:	1821

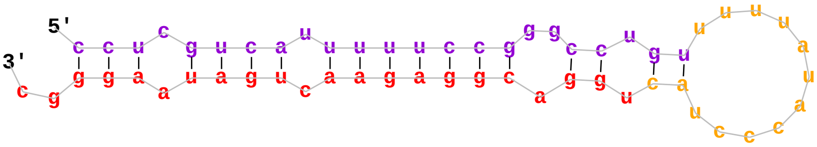

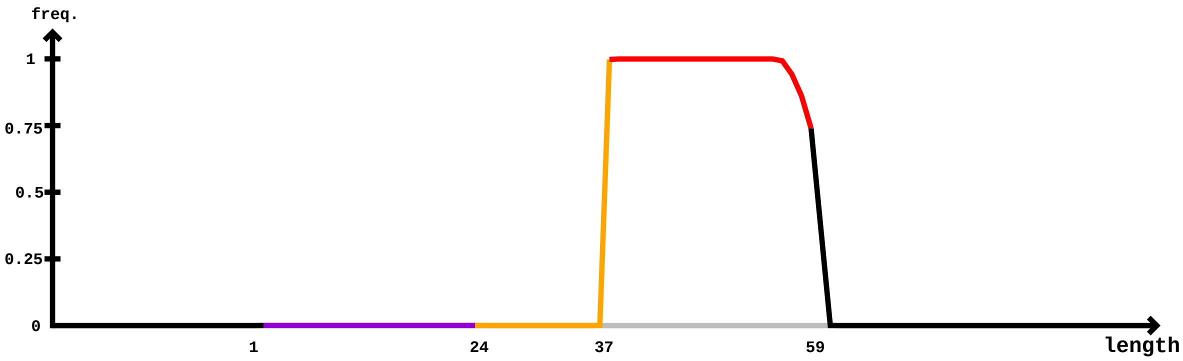

Star **Mature**

```
5'- gaauuuguccgaaagcgggucсucgucauuuuuccgggccuguuuuuauacccuacuggacggagaacugauaagggcucgaauaggaccaagcaacauuugacguuaucugu  -3'   obs
    gaauuuguccgaaagcggguccucgucauuuuuccgggccuguuuuuauacccuacuggacggagaacugauaagggcucgaauaggaccaagcaacauuugacguuaucugu         exp
    ......((((.....((((((((.((((.(((((((..((.((...........)).)).))))))).)))).))))))))....)))).........................    reads   mm   sample
    ....................ccucgucauuuuuccgCgccugu......................................................................    1       1    Fm3
    ....................ccucgucauuuuuccgggccugu......................................................................    14      0    Fm3
    ....................................................cuacuggacggagaacugauaagggc...................................    1       0    Fm3
    .....................................................uacuggacggagaacugauaa.......................................    1       0    Fm3
    .....................................................uacuggacggagaacugauaagggc...................................    1       0    Fm3
    ......................................................acuggacggagaacugaua........................................    2       0    Fm3
    ......................................................acuggacggagaacugauaag......................................    3       0    Fm3
    ......................................................acuggacggagaacugauaagg.....................................    6       0    Fm3
    ......................................................acuggacggagaacugauaagU.....................................    6       1    Fm3
    ......................................................Ucuggacggagaacugauaagg.....................................    1       1    Fm3
    ......................................................Ucuggacggagaacugauaaggg....................................    2       1    Fm3
    ......................................................acuggacggagaacugauaaggU....................................    7       1    Fm3
    ......................................................acuggacggagaacugauaaggA....................................    4       1    Fm3
    ......................................................Ucuggacggagaacugauaagggc...................................    1       1    Fm3
    ......................................................acuggacggagaacugauaagggc...................................    1       0    Fm3
    .......................................................cuggacggagaacugauU........................................    1       1    Fm3
    .......................................................cuggacggagaacugaAa........................................    5       1    Fm3
    .......................................................cuggacggagaacugaua........................................    12      0    Fm3
    .......................................................cuggacggagaacugauaG.......................................    1       1    Fm3
    .......................................................cuggacggagaacugauaa.......................................    16      0    Fm3
    .......................................................Guggacggagaacugauaag......................................    1       1    Fm3
    .......................................................cuggacggagaacugauUag......................................    1       1    Fm3
    .......................................................cuggacggagaacugauaag......................................    103     0    Fm3
    .......................................................cuggacggagaacugauaaA......................................    23      1    Fm3
    .......................................................cuggacggagaacugauaaU......................................    11      1    Fm3
    .......................................................cuggacggagaacugaAaagg.....................................    1       1    Fm3
    .......................................................cuggacggagaacugauGagg.....................................    1       1    Fm3
    .......................................................cuggacggagaacugauaaAg.....................................    1       1    Fm3
    .......................................................cuggacggagaacugauCagg.....................................    2       1    Fm3
    .......................................................cuggacggagaacugauaagU.....................................    9       1    Fm3
    .......................................................cuggacggagaacugaCaagg.....................................    3       1    Fm3
    .......................................................cuggacggagCacugauaagg.....................................    1       1    Fm3
    .......................................................cuggacggagaacugauUagg.....................................    1       1    Fm3
```

Star **Mature**

gaauuuguccgaaagcgggu**ccucgucauuuuuccgggccugu**uuuuauacccuac**uggacggagaacugauaagggc**ucgaauaggaccaagcaacauuugacguuaucugu

....cuggacggagaacugauaagA.....................................	70	1	Fm3
....cuggacggagaacugauaagg.....................................	238	0	Fm3
....cuggacggagGacugauaagg.....................................	1	1	Fm3
....cuggacggagaacugauaaggg....................................	284	0	Fm3
....cuggacggagaacugauaaggU....................................	46	1	Fm3
....cuggacggagaacugauaaggC....................................	3	1	Fm3
....cuggacggagaaAugauaaggg....................................	1	1	Fm3
....cuggacggagaacugaGaaggg....................................	1	1	Fm3
....cuggacggagaacugauGaggg....................................	3	1	Fm3
....cuggacggagaacugauaaggA....................................	67	1	Fm3
....cuggacggagCacugauaaggg....................................	2	1	Fm3
....Nuggacggagaacugauaaggg....................................	1	1	Fm3
....cuggacggagaacugauCaggg....................................	1	1	Fm3
....cuggacggagGacugauaaggg....................................	2	1	Fm3
....cuggacggagaacugauaagggA...................................	64	1	Fm3
....cuggacggagaacugauaagggU...................................	26	1	Fm3
....cuggacggagaacugaAaagggc...................................	1	1	Fm3
....cuggacggGgaacugauaagggc...................................	1	1	Fm3
....cuggacggagaacugaCaagggc...................................	1	1	Fm3
....cuggacggagaacugauaGgggc...................................	2	1	Fm3
....cuggUcggagaacugauaagggc...................................	1	1	Fm3
....cuggacggagaacugauaagggc...................................	212	0	Fm3
....cuggacggagaacugaGaagggcu..................................	1	1	Fm3
....cuggacggagaacugauaagggcu..................................	7	0	Fm3
....uggacggagaacugauaU.......................................	591	1	Fm3
....uggacggagGacugauaa.......................................	15	1	Fm3
....uggacggagaacugaGaa.......................................	5	1	Fm3
....ugAacggagaacugauaa.......................................	5	1	Fm3
....uggacggagaacugUuaa.......................................	5	1	Fm3
....uggacggagaacGgauaa.......................................	1	1	Fm3
....uUgacggagaacugauaa.......................................	4	1	Fm3
....uAgacggagaacugauaa.......................................	14	1	Fm3
....uggacgCagaacugauaa.......................................	1	1	Fm3
....uggacggagaCcugauaa.......................................	13	1	Fm3
....uggUcggagaacugauaa.......................................	1	1	Fm3
....uggacggagaacCgauaa.......................................	3	1	Fm3
....uggacggagaacugauaC.......................................	58	1	Fm3
....uggacggagaacugauGa.......................................	23	1	Fm3
....uggacggaUaacugauaa.......................................	3	1	Fm3
....uggCcggagaacugauaa.......................................	6	1	Fm3
....uggacggagaacuCauaa.......................................	1	1	Fm3
....uggacggagaacugauCa.......................................	41	1	Fm3
....uggacgNagaacugauaa.......................................	1	1	Fm3
....uggacggagaacugaAaa.......................................	1643	1	Fm3
....uggacggaCaacugauaa.......................................	2	1	Fm3
....uggGcggagaacugauaa.......................................	2	1	Fm3
....uggacggagaacugauaa.......................................	18785	0	Fm3
....Aggacggagaacugauaa.......................................	2	1	Fm3
....Nggacggagaacugauaa.......................................	6	1	Fm3
....uggacggagaacuAauaa.......................................	6	1	Fm3
....uggacUgagaacugauaa.......................................	8	1	Fm3
....uggacAgagaacugauaa.......................................	2	1	Fm3
....uggacgUagaacugauaa.......................................	7	1	Fm3
....uggacgAagaacugauaa.......................................	3	1	Fm3
....ugCacggagaacugauaa.......................................	1	1	Fm3
....uggacggUgaacugauaa.......................................	7	1	Fm3
....uggacggagaGcugauaa.......................................	27	1	Fm3
....uggaUggagaacugauaa.......................................	6	1	Fm3
....uggacggGgaacugauaa.......................................	3	1	Fm3
....uggacggCgaacugauaa.......................................	1	1	Fm3
....uggacggagCacugauaa.......................................	2	1	Fm3
....ugUacggagaacugauaa.......................................	9	1	Fm3
....uggaGggagaacugauaa.......................................	1	1	Fm3
....uggacggagaacugGuaa.......................................	1	1	Fm3
....uggacggaAaacugauaa.......................................	2	1	Fm3
....uggacggagaacugauUa.......................................	54	1	Fm3
....Cggacggagaacugauaa.......................................	1	1	Fm3
....uggacggagaacugauaG.......................................	105	1	Fm3
....uCgacggagaacugauaa.......................................	3	1	Fm3
....uggacggagaaUugauaa.......................................	1	1	Fm3

Star Mature

```
gaauuuguccgaaagcggguccucgucauuuuuccgggccuguuuuuauacccuacuggacggagaacugauaagggcucgaauaggaccaagcaacauuugacguuaucugu
........................................................Cggacggagaacugauaag......................................  9      1  Fm3
........................................................uggacggUgaacugauaag......................................  17     1  Fm3
........................................................uggacggagaacugauaag......................................  75493  0  Fm3
........................................................uggacggagaacugGuaag......................................  9      1  Fm3
........................................................uggacNgagaacugauaag......................................  7      1  Fm3
........................................................Nggacggagaacugauaag......................................  30     1  Fm3
........................................................ugNacggagaacugauaag......................................  1      1  Fm3
........................................................uggacggagaacugaAaag......................................  60     1  Fm3
........................................................uggGcggagaacugauaag......................................  17     1  Fm3
........................................................uggacAgagaacugauaag......................................  17     1  Fm3
........................................................uggacggagaacugauaGg......................................  245    1  Fm3
........................................................ugUacggagaacugauaag......................................  27     1  Fm3
........................................................Gggacggagaacugauaag......................................  4      1  Fm3
........................................................ugCacggagaacugauaag......................................  8      1  Fm3
........................................................uggacggagaacugauaUg......................................  256    1  Fm3
........................................................uNgacggagaacugauaag......................................  2      1  Fm3
........................................................uggacggagaaAugauaag......................................  2      1  Fm3
........................................................uggacggagaacugauCag......................................  167    1  Fm3
........................................................uggacggagaacuUauaag......................................  8      1  Fm3
........................................................uggacggaCaacugauaag......................................  2      1  Fm3
........................................................uggaAggagaacugauaag......................................  11     1  Fm3
........................................................uggacggCgaacugauaag......................................  6      1  Fm3
........................................................uggacggaAaacugauaag......................................  4      1  Fm3
........................................................uggacgNagaacugauaag......................................  3      1  Fm3
........................................................ugAacggagaacugauaag......................................  17     1  Fm3
........................................................uggacggagaacugUuaag......................................  10     1  Fm3
........................................................uggacggagaacCgauaag......................................  9      1  Fm3
........................................................uggacUgagaacugauaag......................................  28     1  Fm3
........................................................uggacggagaacGgauaag......................................  1      1  Fm3
........................................................uCgacggagaacugauaag......................................  6      1  Fm3
........................................................uggacggagaacugauaCg......................................  298    1  Fm3
........................................................uggacggagaacugauaaA......................................  48211  1  Fm3
........................................................uggacggagaacugauGag......................................  102    1  Fm3
........................................................uggacggagaacugaCaag......................................  8      1  Fm3
........................................................uggacgAagaacugauaag......................................  6      1  Fm3
........................................................uggacggagaacugCuaag......................................  4      1  Fm3
........................................................uggacggagaacAgauaag......................................  7      1  Fm3
........................................................uAgacggagaacugauaag......................................  53     1  Fm3
........................................................uggaUggagaacugauaag......................................  21     1  Fm3
........................................................uggacggagUacugauaag......................................  18     1  Fm3
........................................................uggacggGgaacugauaag......................................  9      1  Fm3
........................................................uggacCgagaacugauaag......................................  2      1  Fm3
........................................................uggacggagaCcugauaag......................................  116    1  Fm3
........................................................uggacggagaacugauUag......................................  116    1  Fm3
........................................................uggCcggagaacugauaag......................................  11     1  Fm3
........................................................uggacggagGacugauaag......................................  72     1  Fm3
........................................................uggacgCagaacugauaag......................................  7      1  Fm3
........................................................uggacggagaaGugauaag......................................  3      1  Fm3
........................................................Aggacggagaacugauaag......................................  41     1  Fm3
........................................................uggacggagaacuAauaag......................................  10     1  Fm3
........................................................uggacggagaacugauaaC......................................  52     1  Fm3
........................................................uggUcggagaacugauaag......................................  14     1  Fm3
........................................................uggacggagaaUugauaag......................................  23     1  Fm3
........................................................uggacgUagaacugauaag......................................  35     1  Fm3
........................................................uggacggagaacuCauaag......................................  7      1  Fm3
........................................................uUgacggagaacugauaag......................................  37     1  Fm3
........................................................uggaGggagaacugauaag......................................  7      1  Fm3
........................................................uggacggaUaacugauaag......................................  19     1  Fm3
........................................................uggacggagaacugaGaag......................................  2      1  Fm3
........................................................uggacggagaGcugauaag......................................  243    1  Fm3
........................................................uggacggagCacugauaag......................................  5      1  Fm3
........................................................uggacggagaacugauaaU......................................  15180  1  Fm3
........................................................uggacggagaacGgauaagg.....................................  7      1  Fm3
........................................................Gggacggagaacugauaagg.....................................  16     1  Fm3
........................................................uggacggagCacugauaagg.....................................  14     1  Fm3
........................................................uAgacggagaacugauaagg.....................................  79     1  Fm3
........................................................uggacggagaacugauCagg.....................................  354    1  Fm3
........................................................uNgacggagaacugauaagg.....................................  1      1  Fm3
........................................................uggacggagaacuAauaagg.....................................  33     1  Fm3
........................................................ugAacggagaacugauaagg.....................................  36     1  Fm3
```

```
                    Star                              Mature
gaauuuguccgaaagcggguccucgucauuuuuccgggccuguuuuuauacccuacuggacggagaacugauaagggcucgaauaggaccaagcaacauuugacguuaucugu
........................................................ugNacggagaacugauaagg.....................................    1       1    Fm3
........................................................uggacggagaacugauaagA.....................................    43430   1    Fm3
........................................................uggacggagaacuUauaagg.....................................    17      1    Fm3
........................................................uggacgUagaacugauaagg.....................................    46      1    Fm3
........................................................uggacgNagaacugauaagg.....................................    9       1    Fm3
........................................................uggacggagaacugauUagg.....................................    249     1    Fm3
........................................................uggacggagaacAgauaagg.....................................    4       1    Fm3
........................................................uggacggagaacugauGagg.....................................    266     1    Fm3
........................................................ugUacggagaacugauaagg.....................................    42      1    Fm3
........................................................uggacgCagaacugauaagg.....................................    11      1    Fm3
........................................................uggacggagaacCgauaagg.....................................    19      1    Fm3
........................................................uggacggagUacugauaagg.....................................    32      1    Fm3
........................................................uggacggagGacugauaagg.....................................    235     1    Fm3
........................................................uggacggagaaUugauaagg.....................................    31      1    Fm3
........................................................uggaGggagaacugauaagg.....................................    8       1    Fm3
........................................................uggacggaCaacugauaagg.....................................    11      1    Fm3
........................................................uggacggagaacugauaagC.....................................    22      1    Fm3
........................................................Nggacggagaacugauaagg.....................................    83      1    Fm3
........................................................uggacgAagaacugauaagg.....................................    12      1    Fm3
........................................................uggacggagaacugauaagg.....................................    154018  0    Fm3
........................................................ugCacggagaacugauaagg.....................................    13      1    Fm3
........................................................uCgacggagaacugauaagg.....................................    18      1    Fm3
........................................................uggacggagaacugauaagU.....................................    5388    1    Fm3
........................................................uggCcggagaacugauaagg.....................................    34      1    Fm3
........................................................uggacggagaacugauaGgg.....................................    663     1    Fm3
........................................................uggacggagaacugaCaagg.....................................    19      1    Fm3
........................................................uggGcggagaacugauaagg.....................................    36      1    Fm3
........................................................uggacAgagaacugauaagg.....................................    37      1    Fm3
........................................................uggacggCgaacugauaagg.....................................    9       1    Fm3
........................................................uggacggagaacugauaCgg.....................................    648     1    Fm3
........................................................Cggacggagaacugauaagg.....................................    26      1    Fm3
........................................................uggacggagaacugauaaUg.....................................    45      1    Fm3
........................................................uggacggaUaacugauaagg.....................................    32      1    Fm3
........................................................uggacUgagaacugauaagg.....................................    50      1    Fm3
........................................................uggUcggagaacugauaagg.....................................    25      1    Fm3
........................................................uggacggUgaacugauaagg.....................................    37      1    Fm3
........................................................Aggacggagaacugauaagg.....................................    638     1    Fm3
........................................................uggacggagaacuCauaagg.....................................    9       1    Fm3
........................................................uggacggagaaAugauaagg.....................................    10      1    Fm3
........................................................uggacggagaCcugauaagg.....................................    1074    1    Fm3
........................................................uggacCgagaacugauaagg.....................................    6       1    Fm3
........................................................uggaAggagaacugauaagg.....................................    20      1    Fm3
........................................................uggacggagaacugauaUgg.....................................    476     1    Fm3
........................................................uggacggagaacugauaaAg.....................................    936     1    Fm3
........................................................uggaUggagaacugauaagg.....................................    57      1    Fm3
........................................................uggacggagaacugGuaagg.....................................    15      1    Fm3
........................................................uggacggagaacugaGaagg.....................................    6       1    Fm3
........................................................uggacggagaGcugauaagg.....................................    1812    1    Fm3
........................................................uggacggaAaacugauaagg.....................................    10      1    Fm3
........................................................uggacggagaacugaAaagg.....................................    7       1    Fm3
........................................................uUgacggagaacugauaagg.....................................    57      1    Fm3
........................................................uggacggagaacugauaaCg.....................................    5       1    Fm3
........................................................uggacggagaacugCuaagg.....................................    10      1    Fm3
........................................................uggacNgagaacugauaagg.....................................    17      1    Fm3
........................................................uggacggGgaacugauaagg.....................................    22      1    Fm3
........................................................uggacggagaaGugauaagg.....................................    4       1    Fm3
........................................................uggacggagaacugUuaagg.....................................    33      1    Fm3
........................................................uggacggagaacugauaGggg....................................    1166    1    Fm3
........................................................uggacggaAaacugauaaggg....................................    13      1    Fm3
........................................................uggacggUgaacugauaaggg....................................    90      1    Fm3
........................................................uggacggagaacugaAaaggg....................................    13      1    Fm3
........................................................uggacggagaacuUauaaggg....................................    25      1    Fm3
........................................................uggacggagaacGgauaaggg....................................    11      1    Fm3
........................................................uggacCgagaacugauaaggg....................................    7       1    Fm3
........................................................uggacggagUacugauaaggg....................................    41      1    Fm3
........................................................uggacgCagaacugauaaggg....................................    12      1    Fm3
........................................................uggacggagaacugauaaggg....................................    200595  0    Fm3
........................................................uNgacggagaacugauaaggg....................................    3       1    Fm3
........................................................uggacggagaacugauaagCg....................................    15      1    Fm3
........................................................uCgacggagaacugauaaggg....................................    18      1    Fm3
```

Star Mature

```
gaauuuguccgaaagcgggucCucgucauuuuuccgggccuguuuuuauacccuacuggacggagaacugauaagggcucgaauaggaccaagcaacauuugacguuaucugu
........................................................ugCacggagaacugauaaggg....................................  14      1  Fm3
........................................................uggacggagaacugGuaaggg....................................  33      1  Fm3
........................................................uggacggagaCcugauaaggg....................................  674     1  Fm3
........................................................uggacggagCacugauaaggg....................................  17      1  Fm3
........................................................uggacggagaacugauUaggg....................................  327     1  Fm3
........................................................uggCcggagaacugauaaggg....................................  29      1  Fm3
........................................................ugUacggagaacugauaaggg....................................  57      1  Fm3
........................................................uggacggaCaacugauaaggg....................................  8       1  Fm3
........................................................uggacggagaacugauaUggg....................................  709     1  Fm3
........................................................ugAacggagaacugauaaggg....................................  49      1  Fm3
........................................................uggacggagaaGugauaaggg....................................  6       1  Fm3
........................................................uggacggagaacAgauaaggg....................................  10      1  Fm3
........................................................uggUcggagaacugauaaggg....................................  33      1  Fm3
........................................................uggaUggagaacugauaaggg....................................  66      1  Fm3
........................................................uggacNgagaacugauaaggg....................................  30      1  Fm3
........................................................Cggacggagaacugauaaggg....................................  31      1  Fm3
........................................................Gggacggagaacugauaaggg....................................  12      1  Fm3
........................................................uggacggagaaAugauaaggg....................................  6       1  Fm3
........................................................uggacggagaacugauaaggA....................................  98094   1  Fm3
........................................................uUgacggagaacugauaaggg....................................  58      1  Fm3
........................................................uggacggagaacugauaaUgg....................................  103     1  Fm3
........................................................uggacggagaacugauCaggg....................................  465     1  Fm3
........................................................uggacggagaacugCuaaggg....................................  11      1  Fm3
........................................................uggacggagaaUugauaaggg....................................  50      1  Fm3
........................................................uggacggagaacugauaaggU....................................  7452    1  Fm3
........................................................ugNacggagaacugauaaggg....................................  1       1  Fm3
........................................................uggacUgagaacugauaaggg....................................  73      1  Fm3
........................................................uggacggGgaacugauaaggg....................................  44      1  Fm3
........................................................uggacggaUaacugauaaggg....................................  41      1  Fm3
........................................................uggGcggagaacugauaaggg....................................  44      1  Fm3
........................................................uggacggagGacugauaaggg....................................  195     1  Fm3
........................................................uggacggCgaacugauaaggg....................................  14      1  Fm3
........................................................uggacggagaacugauaagAg....................................  189     1  Fm3
........................................................uggacggagaacuCauaaggg....................................  14      1  Fm3
........................................................uggacgAagaacugauaaggg....................................  13      1  Fm3
........................................................uggacgNagaacugauaaggg....................................  10      1  Fm3
........................................................uggacAgagaacugauaaggg....................................  34      1  Fm3
........................................................uggacggagaacugaCaaggg....................................  20      1  Fm3
........................................................uggacgUagaacugauaaggg....................................  63      1  Fm3
........................................................Nggacggagaacugauaaggg....................................  111     1  Fm3
........................................................uggacggagaacugauaCggg....................................  780     1  Fm3
........................................................uggacggagaacugauaagUg....................................  79      1  Fm3
........................................................uggacggagaacugauaaCgg....................................  5       1  Fm3
........................................................uggacggagaacugauaaAgg....................................  22      1  Fm3
........................................................uggacggagaGcugauaaggg....................................  1292    1  Fm3
........................................................uggacggagaacugauGaggg....................................  294     1  Fm3
........................................................uggacggagaacugaGaaggg....................................  4       1  Fm3
........................................................uggacggagaacCgauaaggg....................................  22      1  Fm3
........................................................Aggacggagaacugauaaggg....................................  389     1  Fm3
........................................................uggaGggagaacugauaaggg....................................  5       1  Fm3
........................................................uggacggagaacugUuaaggg....................................  48      1  Fm3
........................................................uAgacggagaacugauaaggg....................................  98      1  Fm3
........................................................uggaAggagaacugauaaggg....................................  20      1  Fm3
........................................................uggacggagaacugauaaggC....................................  78      1  Fm3
........................................................uggacggagaacuAauaaggg....................................  52      1  Fm3
........................................................uggacggagGacugauaagggc...................................  581     1  Fm3
........................................................uggacggagaacugUuaagggc...................................  135     1  Fm3
........................................................uggacggagaacugauaagggU...................................  12581   1  Fm3
........................................................uggacggagaacugauaaAggc...................................  51      1  Fm3
........................................................Gggacggagaacugauaagggc...................................  40      1  Fm3
........................................................uggacggagaGcugauaagggc...................................  1460    1  Fm3
........................................................uggacggagaacuCauaagggc...................................  58      1  Fm3
........................................................uggUcggagaacugauaagggc...................................  147     1  Fm3
........................................................uggacggagaacugauaagCgc...................................  49      1  Fm3
........................................................uCgacggagaacugauaagggc...................................  85      1  Fm3
........................................................uggacggagaacugauaagggc...................................  790111  0  Fm3
........................................................uggacggagaaUugauaagggc...................................  189     1  Fm3
........................................................uggacggagaacGgauaagggc...................................  25      1  Fm3
........................................................ugCacggagaacugauaagggc...................................  61      1  Fm3
........................................................uggacggagaacCgauaagggc...................................  90      1  Fm3
```

Star Mature

```
gaauuuguccgaaagcggguccucgucauuuuuccgggccuguuuuuauacccuacuggacggagaacugauaagggcucgaauaggaccaagcaacauuugacguuaucugu
........................................................uggacggCgaacugauaagggc...................................  42  1  Fm3
........................................................uggacUgagaacugauaagggc...................................  245  1  Fm3
........................................................uggacggagaacugaGaagggc...................................  19  1  Fm3
........................................................uggacggagaacugauaCgggc...................................  3025  1  Fm3
........................................................uggaGggagaacugauaagggc...................................  32  1  Fm3
........................................................ugAacggagaacugauaagggc...................................  192  1  Fm3
........................................................uggacggagaacugauGagggc...................................  1829  1  Fm3
........................................................uggacAgagaacugauaagggc...................................  155  1  Fm3
........................................................uggacggaUaacugauaagggc...................................  182  1  Fm3
........................................................uggacggagaacugCuaagggc...................................  28  1  Fm3
........................................................uggCcggagaacugauaagggc...................................  161  1  Fm3
........................................................uNgacggagaacugauaagggc...................................  10  1  Fm3
........................................................uggacggagaacugauUagggc...................................  911  1  Fm3
........................................................uggacggagaacugauaaNggc...................................  1  1  Fm3
........................................................uggacggagaaGugauaagggc...................................  15  1  Fm3
........................................................uggacggagaacugauaagggA...................................  100006  1  Fm3
........................................................uggacggagCacugauaagggc...................................  55  1  Fm3
........................................................uggacggagaacugGuaagggc...................................  95  1  Fm3
........................................................uggacCgagaacugauaagggc...................................  40  1  Fm3
........................................................uggacgCagaacugauaagggc...................................  46  1  Fm3
........................................................Aggacggagaacugauaagggc...................................  237  1  Fm3
........................................................Nggacggagaacugauaagggc...................................  405  1  Fm3
........................................................uggacggagaacugaAaagggc...................................  29  1  Fm3
........................................................uggacggagaacuUauaagggc...................................  83  1  Fm3
........................................................uggacggagUacugauaagggc...................................  123  1  Fm3
........................................................uggacggagaacugauaGgggc...................................  5830  1  Fm3
........................................................uggacggagaCcugauaagggc...................................  696  1  Fm3
........................................................uggacggagaacugaCaagggc...................................  82  1  Fm3
........................................................uggacggUgaacugauaagggc...................................  214  1  Fm3
........................................................uggacggaAaacugauaagggc...................................  51  1  Fm3
........................................................uAgacggagaacugauaagggc...................................  387  1  Fm3
........................................................uggacggagaacugauaaCggc...................................  38  1  Fm3
........................................................uggacggagaacugauaagAgc...................................  104  1  Fm3
........................................................uggacggagaacAgauaagggc...................................  51  1  Fm3
........................................................uUgacggagaacugauaagggc...................................  256  1  Fm3
........................................................ugNacggagaacugauaagggc...................................  1  1  Fm3
........................................................uggacggagaacugauNagggc...................................  3  1  Fm3
........................................................uggacggagaacugauaagggG...................................  97  1  Fm3
........................................................uggGcggagaacugauaagggc...................................  198  1  Fm3
........................................................uggacggagaacugauaaggUc...................................  273  1  Fm3
........................................................uggacggGgaacugauaagggc...................................  180  1  Fm3
........................................................uggacggagaacuAauaagggc...................................  167  1  Fm3
........................................................uggacggagaacugauCagggc...................................  1554  1  Fm3
........................................................ugUacggagaacugauaagggc...................................  244  1  Fm3
........................................................uggacggagaacugauaaUggc...................................  308  1  Fm3
........................................................uggacggagaacugauaUgggc...................................  2622  1  Fm3
........................................................uggacggagaacugauaagUgc...................................  281  1  Fm3
........................................................uggacgUagaacugauaagggc...................................  263  1  Fm3
........................................................uggacggaCaacugauaagggc...................................  45  1  Fm3
........................................................uggacggagaaAugauaagggc...................................  34  1  Fm3
........................................................uggaAggagaacugauaagggc...................................  91  1  Fm3
........................................................Cggacggagaacugauaagggc...................................  141  1  Fm3
........................................................uggacgAagaacugauaagggc...................................  78  1  Fm3
........................................................uggacgNagaacugauaagggc...................................  41  1  Fm3
........................................................uggacggagaacugauaaggAc...................................  157  1  Fm3
........................................................uggacggagaacugauaaggCc...................................  71  1  Fm3
........................................................uggacNgagaacugauaagggc...................................  104  1  Fm3
........................................................uggaUggagaacugauaagggc...................................  278  1  Fm3
........................................................uggacggagaacuCauaagggcu.................................  1  1  Fm3
........................................................uggacggagaacCgauaagggcu.................................  3  1  Fm3
........................................................uggacNgagaacugauaagggcu.................................  3  1  Fm3
........................................................uggacggagaacugauaagUgcu.................................  4  1  Fm3
........................................................uggacggagaacugUuaagggcu.................................  3  1  Fm3
........................................................uggCcggagaacugauaagggcu.................................  5  1  Fm3
........................................................uggacggagaacugauaaUggcu.................................  6  1  Fm3
........................................................uggacgUagaacugauaagggcu.................................  7  1  Fm3
........................................................uggacggagaCcugauaagggcu.................................  15  1  Fm3
........................................................uggacggagaaGugauaagggcu.................................  1  1  Fm3
........................................................uggacggagaacugauCagggcu.................................  32  1  Fm3
........................................................uggacggagaacugauaagggcG..................................  246  1  Fm3
```

```
                   Star                                Mature
gaauuuguccgaaagcggguccucgucauuuuuccgggccuguuuuuauacccuacuggacggagaacugauaagggcucgaauaggaccaagcaacauuugacguuaucugu
........................................................uggacggagaacAgauaagggcu..................................   1      1   Fm3
........................................................uggacUgagaacugauaagggcu..................................   1      1   Fm3
........................................................uggacggagaacugauaCgggcu..................................   73     1   Fm3
........................................................uggUcggagaacugauaagggcu..................................   6      1   Fm3
........................................................uggacggagaacugCuaagggcu..................................   1      1   Fm3
........................................................uggacggagaacugauaaAggcu..................................   3      1   Fm3
........................................................ugUacggagaacugauaagggcu..................................   6      1   Fm3
........................................................uggacggagGacugauaagggcu..................................   20     1   Fm3
........................................................uCgacggagaacugauaagggcu..................................   2      1   Fm3
........................................................uAgacggagaacugauaagggcu..................................   12     1   Fm3
........................................................uggacggagaaAugauaagggcu..................................   1      1   Fm3
........................................................uggacggagaacugauaagggGu..................................   12     1   Fm3
........................................................uggacggaCaacugauaagggcu..................................   1      1   Fm3
........................................................uggacggagaGcugauaagggcu..................................   35     1   Fm3
........................................................uggacAgagaacugauaagggcu..................................   6      1   Fm3
........................................................Nggacggagaacugauaagggcu..................................   10     1   Fm3
........................................................uUgacggagaacugauaagggcu..................................   6      1   Fm3
........................................................uggacggagaacugauaagggUu..................................   1283   1   Fm3
........................................................uggacggGgaacugauaagggcu..................................   5      1   Fm3
........................................................uggacggUgaacugauaagggcu..................................   5      1   Fm3
........................................................uggacggagaacugauaagAgcu..................................   2      1   Fm3
........................................................uggacggagaacugauaaggAcu..................................   3      1   Fm3
........................................................uggacggagaaUugauaagggcu..................................   10     1   Fm3
........................................................ugAacggagaacugauaagggcu..................................   5      1   Fm3
........................................................uggacggagaacugaAaagggcu..................................   1      1   Fm3
........................................................Aggacggagaacugauaagggcu..................................   2      1   Fm3
........................................................uggacggaUaacugauaagggcu..................................   4      1   Fm3
........................................................uggacggagaacuUauaagggcu..................................   1      1   Fm3
........................................................uggacggagaacugauaaggCcu..................................   2      1   Fm3
........................................................uggacggagaacugauaagggAu..................................   5158   1   Fm3
........................................................Cggacggagaacugauaagggcu..................................   1      1   Fm3
........................................................uggaUggagaacugauaagggcu..................................   9      1   Fm3
........................................................uggacggagaacugauaaggUcu..................................   7      1   Fm3
........................................................uggacggagaacugGuaagggcu..................................   3      1   Fm3
........................................................uggacggagaacugauaagggcu..................................   17693  0   Fm3
........................................................uggacggagaacugauaUgggcu..................................   46     1   Fm3
........................................................uggGcggagaacugauaagggcu..................................   4      1   Fm3
........................................................uggaAggagaacugauaagggcu..................................   1      1   Fm3
........................................................uggacggagCacugauaagggcu..................................   2      1   Fm3
........................................................uggacggagaacugauaagggcC..................................   86     1   Fm3
........................................................uggacggagaacugauaagCgcu..................................   1      1   Fm3
........................................................uggacggagaacugauaGgggcu..................................   110    1   Fm3
........................................................uggacggagUacugauaagggcu..................................   4      1   Fm3
........................................................uggacggCgaacugauaagggcu..................................   1      1   Fm3
........................................................uggacggagaacuAauaagggcu..................................   3      1   Fm3
........................................................uggacggagaacugauGagggcu..................................   34     1   Fm3
........................................................uggacggagaacugauUagggcu..................................   24     1   Fm3
........................................................uggacggagaacugauaagggcuc.................................   4      0   Fm3
........................................................uggacggagaacugauaagggcuG.................................   3      1   Fm3
........................................................uggacggagaacugauaagggcuA.................................   60     1   Fm3
........................................................uggacggagaacugauaagggcAc.................................   9      1   Fm3
.........................................................ggacggaAaacugauaag......................................   1      1   Fm3
.........................................................ggacggagaacugauCag......................................   1      1   Fm3
.........................................................ggacggagaacugaGaag......................................   1      1   Fm3
.........................................................Ugacggagaacugauaag......................................   6      1   Fm3
.........................................................ggacggagaacugaAaag......................................   1      1   Fm3
.........................................................ggacggagaacugauaCg......................................   1      1   Fm3
.........................................................ggacggagaacugauaag......................................   135    0   Fm3
.........................................................ggacggagaacugauaaA......................................   68     1   Fm3
.........................................................ggaUggagaacugauaag......................................   1      1   Fm3
.........................................................ggacggagaacugauaaC......................................   1      1   Fm3
.........................................................ggacggagCacugauaag......................................   1      1   Fm3
.........................................................ggacggagaacugauaaU......................................   21     1   Fm3
.........................................................ggGcggagaacugauaag......................................   1      1   Fm3
.........................................................ggacggCgaacugauaagg.....................................   1      1   Fm3
.........................................................ggacggagaCcugauaagg.....................................   7      1   Fm3
.........................................................ggacggaAaacugauaagg.....................................   1      1   Fm3
.........................................................ggacggagaacugauaagU.....................................   8      1   Fm3
.........................................................ggacggagaaAugauaagg.....................................   3      1   Fm3
.........................................................ggacggagaacugauaagA.....................................   108    1   Fm3
```

Star **Mature**

gaauuuguccgaaagcggguccucgucauuuuuccgggccuguuuuuauacccuacuggacggagaacugauaagggcucgaauaggaccaagcaacauuugacguuaucugu			
....ggacggaCaacugauaagg.....................................	1	1	Fm3
....ggacggagaacCgauaagg.....................................	2	1	Fm3
....ggGcggagaacugauaagg.....................................	1	1	Fm3
....ggacggagaacugauaagg.....................................	317	0	Fm3
....ggacggagaacugauaUgg.....................................	1	1	Fm3
....ggacggagaacugaAaagg.....................................	1	1	Fm3
....ggacggagaacugauaGgg.....................................	2	1	Fm3
....ggacggagaacugauaaAg.....................................	1	1	Fm3
....ggaAggagaacugauaagg.....................................	1	1	Fm3
....ggacggagaacGgauaagg.....................................	1	1	Fm3
....Ugacggagaacugauaagg.....................................	9	1	Fm3
....ggacggagaGcugauaagg.....................................	6	1	Fm3
....ggacggagaacugauaaUg.....................................	2	1	Fm3
....Ngacggagaacugauaagg.....................................	1	1	Fm3
....ggacggagaacugauCagg.....................................	3	1	Fm3
....ggacggagaaUugauaagg.....................................	1	1	Fm3
....ggacggagaacugauaaggC....................................	5	1	Fm3
....ggacggagaacGgauaaggg....................................	1	1	Fm3
....ggacggagaacugaAaaggg....................................	1	1	Fm3
....ggacggagCacugauaaggg....................................	1	1	Fm3
....ggacggagaacugauCaggg....................................	5	1	Fm3
....ggacggagaacugUuaaggg....................................	1	1	Fm3
....ggacggagaaAugauaaggg....................................	1	1	Fm3
....ggacgAagaacugauaaggg....................................	1	1	Fm3
....ggacggagaacugauaaggg....................................	386	0	Fm3
....ggacggagaacCgauaaggg....................................	2	1	Fm3
....ggacggagaacugauaaggU....................................	16	1	Fm3
....ggacggagaacugauaCggg....................................	3	1	Fm3
....ggacggagaacugauaGggg....................................	3	1	Fm3
....ggacggagaacugauUaggg....................................	2	1	Fm3
....Ugacggagaacugauaaggg....................................	14	1	Fm3
....ggacggagaacugauaaggA....................................	253	1	Fm3
....ggacggagaGcugauaaggg....................................	4	1	Fm3
....ggacggagaCcugauaaggg....................................	5	1	Fm3
....ggacggagaaGugauaaggg....................................	2	1	Fm3
....ggacggUgaacugauaaggg....................................	1	1	Fm3
....ggacggagaacugGuaaggg....................................	1	1	Fm3
....ggacggagUacugauaaggg....................................	1	1	Fm3
....ggacggagaacugauaaAgg....................................	1	1	Fm3
....ggacggagaacuCauaagggc...................................	1	1	Fm3
....ggCcggagaacugauaagggc...................................	1	1	Fm3
....ggacggagaacugauaagggA...................................	230	1	Fm3
....ggacggagaGcugauaagggc...................................	3	1	Fm3
....ggacggagaacugauGagggc...................................	2	1	Fm3
....ggacggagaacCgauaagggc...................................	1	1	Fm3
....ggacCgagaacugauaagggc...................................	3	1	Fm3
....ggacggagaacugaGaagggc...................................	2	1	Fm3
....ggacggagaacAgauaagggc...................................	1	1	Fm3
....ggaAggagaacugauaagggc...................................	4	1	Fm3
....Ngacggagaacugauaagggc...................................	1	1	Fm3
....ggacggagaacugauaaggCc...................................	7	1	Fm3
....ggacggagaacugauaCgggc...................................	3	1	Fm3
....ggacggagaacuUauaagggc...................................	2	1	Fm3
....ggacggagaacugauUagggc...................................	2	1	Fm3
....ggacggagaacugUuaagggc...................................	1	1	Fm3
....ggacggagaacugauaGgggc...................................	7	1	Fm3
....ggacggagUacugauaagggc...................................	2	1	Fm3
....ggacggagaacugauaUgggc...................................	1	1	Fm3
....ggacggagaacugauaagggU...................................	23	1	Fm3
....ggacggagaacGgauaagggc...................................	6	1	Fm3
....ggacggagaacugauaaUggc...................................	1	1	Fm3
....ggacggaCaacugauaagggc...................................	1	1	Fm3
....ggacggagaacugauaaAggc...................................	2	1	Fm3
....ggaGggagaacugauaagggc...................................	1	1	Fm3
....ggacggUgaacugauaagggc...................................	1	1	Fm3
....ggacggagaaGugauaagggc...................................	4	1	Fm3
....ggacggagGacugauaagggc...................................	1	1	Fm3
....ggacggagaacugauaagCgc...................................	1	1	Fm3
....ggacggagaacugauaaggAc...................................	8	1	Fm3
....ggacggagCacugauaagggc...................................	15	1	Fm3

Star **Mature**

```
gaauuuguccgaaagcgguccucgucauuuuuccgggccuguuuuuauacccuacuggacggagaacugauaagggcucgaauaggaccaagcaacauuugacguuaucugu
........................................................ggGcggagaacugauaagggc...................................   3     1  Fm3
........................................................ggacggagaaUugauaagggc...................................   3     1  Fm3
........................................................ggacggagaaAugauaagggc...................................   7     1  Fm3
........................................................ggacggCgaacugauaagggc...................................   6     1  Fm3
........................................................ggacggagaacugauaagAgc...................................   3     1  Fm3
........................................................ggacggagaacugaAaagggc...................................   1     1  Fm3
........................................................Ugacggagaacugauaagggc...................................   34    1  Fm3
........................................................ggacggaAaacugauaagggc...................................   2     1  Fm3
........................................................ggacggagaacugauaaggUc...................................   6     1  Fm3
........................................................ggacggagaCcugauaagggc...................................   4     1  Fm3
........................................................ggacggagaacugauaagggc...................................   1615  0  Fm3
........................................................ggacggagaacugauaagggAu..................................   10    1  Fm3
........................................................ggacggagaacugauaagggcu..................................   26    0  Fm3
........................................................ggacggagaacugauaagggUu..................................   3     1  Fm3
........................................................Ugacggagaacugauaagggcu..................................   2     1  Fm3
........................................................ggacggagaacugauaagggcAc.................................   1     1  Fm3
.........................................................Uacggagaacugauaaggg....................................   3     1  Fm3
.........................................................gacggagaacugauaaggg....................................   3     0  Fm3
.........................................................gacggagaaUugauaagggc...................................   2     1  Fm3
.........................................................gacggagaacugauaagggA...................................   1     1  Fm3
.........................................................Uacggagaacugauaagggc...................................   6     1  Fm3
.........................................................gacggagaacugauaagggc...................................   9     0  Fm3
..........................................................acggagaacugauaagggc...................................   1     0  Fm3
..........................................................acggagaacugauaagggcu..................................   1     0  Fm3
...........................................................Uggagaacugauaagggc...................................   10    1  Fm3
...........................................................cggagaacugauaagggc...................................   1     0  Fm3
...........................................................Uggagaacugauaagggcu..................................   1     1  Fm3

..................uccucgucauuuuuccgggccugu......................................................................   3     0  Fm1
...................ccucgucauuuuuccggg...........................................................................   4     0  Fm1
...................ccucgucauuuuuccgggcc.........................................................................   2     0  Fm1
...................ccucgucauuuuuccggCccu........................................................................   1     1  Fm1
...................ccucgucauuuuuccgggccu........................................................................   5     0  Fm1
...................ccucgucauuuuuccgggccug.......................................................................   9     0  Fm1
...................ccucgucauuuuuccgggccGg.......................................................................   1     1  Fm1
...................ccucgucauuuuuccgggccugA......................................................................   6     1  Fm1
...................ccAcgucauuuuuccgggccugu......................................................................   1     1  Fm1
...................ccucgucauuuuCccgggccugu......................................................................   2     1  Fm1
...................ccucgucauuuuuccgggcUugu......................................................................   1     1  Fm1
...................ccucgucauuuuuccggUccugu......................................................................   6     1  Fm1
...................Ucucgucauuuuuccgggccugu......................................................................   7     1  Fm1
...................ccGcgucauuuuuccgggccugu......................................................................   1     1  Fm1
...................ccucgucauuuuuccgCgccugu......................................................................   2     1  Fm1
...................ccucgucauuuuuccgggccugG......................................................................   20    1  Fm1
...................ccUgucauuuuuccgggccugu.......................................................................   2     1  Fm1
...................ccucgucauuuuuccgggccCgu......................................................................   1     1  Fm1
...................Ncucgucauuuuuccgggccugu......................................................................   1     1  Fm1
...................ccucgucauuuuuccgAgccugu......................................................................   1     1  Fm1
...................ccucgucauuuuuccgUgccugu......................................................................   3     1  Fm1
...................ccucgucauuuuuccggAccugu......................................................................   1     1  Fm1
...................ccucgucauuuuuccUggccugu......................................................................   1     1  Fm1
...................ccucgucauuGuuccgggccugu......................................................................   1     1  Fm1
...................ccucgucauuuuuccggCccugu......................................................................   2     1  Fm1
...................ccucgucauuuuuccgggccugC......................................................................   1     1  Fm1
...................ccucgucGuuuuuccgggccugu......................................................................   1     1  Fm1
...................ccucgucauuuuuccgggccugu......................................................................   730   0  Fm1
...................ccucgucauuAuuccgggccugu......................................................................   1     1  Fm1
...................ccucgucaAuuuuccgggccugu......................................................................   1     1  Fm1
...................ccucgucauuuuUcgggccugu.......................................................................   1     1  Fm1
...................ccucgucauuCuuccgggccugu......................................................................   1     1  Fm1
...................Ucucgucauuuuuccgggccuguu.....................................................................   2     1  Fm1
...................ccucgucauuuuuccgggccuguA.....................................................................   3     1  Fm1
...................ccucgucauuuuuccgggccuguu.....................................................................   75    0  Fm1
...................ccucgucauuuuuccgggccuguuu....................................................................   16    0  Fm1
...................ccucgucauuuuuccgggccuguuuu...................................................................   2     0  Fm1
....................cucgucauuuuuccgggccugu......................................................................   17    0  Fm1
....................cucgucauuuuuccgggccuguu.....................................................................   2     0  Fm1
....................cucgucauuuuuccgggccuguuu....................................................................   4     0  Fm1
......................cgucauuuuuccgggccugu......................................................................   2     0  Fm1
...........................................uuuauacccuacuggacggagaacugau.........................................   1     0  Fm1
```

```
                    Star                                   Mature
gaauuuguccgaaagcggguccucgucauuuuuccgggccuguuuuuauacccuacuggacggagaacugauaagggcucgaauaggaccaagcaacauuugacguuaucugu
......................................................acuggacggagaacugauaaU......................................	4	1	Fm1
......................................................acuggacggagaacugauaagg.....................................	2	0	Fm1
......................................................acuggacggagaacugauaaggA....................................	2	1	Fm1
......................................................acuggacggagaacugauaagggc...................................	3	0	Fm1
.......................................................cuggacggagaacugaua........................................	2	0	Fm1
.......................................................cuggacggagaacugaAaa.......................................	5	1	Fm1
.......................................................cuggacggagaacugauCa.......................................	1	1	Fm1
.......................................................cuggacggagaacugauaa.......................................	6	0	Fm1
.......................................................cuggacggagGacugauaa.......................................	1	1	Fm1
.......................................................cuggacggagaacugauaaU......................................	3	1	Fm1
.......................................................cuggacggagaacugauaag......................................	36	0	Fm1
.......................................................cuggacggagaacugauaaA......................................	14	1	Fm1
.......................................................cAggacggagaacugauaag......................................	1	1	Fm1
.......................................................Nuggacggagaacugauaagg.....................................	1	1	Fm1
.......................................................cuggacggagaacugauaagU.....................................	1	1	Fm1
.......................................................cuggacggagaacugauaagA.....................................	18	1	Fm1
.......................................................cuggacggagaacAgauaagg.....................................	1	1	Fm1
.......................................................cuggacggaUaacugauaagg.....................................	1	1	Fm1
.......................................................cuggacggagaacugauCagg.....................................	1	1	Fm1
.......................................................cuggacggagaacugauaagg.....................................	165	0	Fm1
.......................................................cuggaNggagaacugauaagg.....................................	1	1	Fm1
.......................................................cuggacUgagaacugauaaggg....................................	1	1	Fm1
.......................................................cuggacggagaacugauaaggU....................................	32	1	Fm1
.......................................................cuggacggagaacugauGaggg....................................	2	1	Fm1
.......................................................cuggacggagCacugauaaggg....................................	1	1	Fm1
.......................................................cuggacggagaacugauaaggA....................................	15	1	Fm1
.......................................................cuggacggagaacugaGaaggg....................................	1	1	Fm1
.......................................................cuggacggagaacugauaaggC....................................	5	1	Fm1
.......................................................cuggacggagaacugauaaggg....................................	169	0	Fm1
.......................................................cuggacggagUacugauaaggg....................................	1	1	Fm1
.......................................................cuggacUgagaacugauaagggc...................................	1	1	Fm1
.......................................................cuggacggagaacugauaagggU...................................	4	1	Fm1
.......................................................cuggacggagaacugauCagggc...................................	1	1	Fm1
.......................................................cuggacggGgaacugauaagggc...................................	2	1	Fm1
.......................................................cuggacAgagaacugauaagggc...................................	1	1	Fm1
.......................................................cuggacggagUacugauaagggc...................................	2	1	Fm1
.......................................................cuggacggagaacugaAaagggc...................................	1	1	Fm1
.......................................................cuggacggagaacugauGagggc...................................	1	1	Fm1
.......................................................cuggacggUgaacugauaagggc...................................	1	1	Fm1
.......................................................cGggacggagaacugauaagggc...................................	1	1	Fm1
.......................................................cuggacggagaaUugauaagggc...................................	1	1	Fm1
.......................................................cuggacggagGacugauaagggc...................................	4	1	Fm1
.......................................................cuggacggagaacugaCaagggc...................................	4	1	Fm1
.......................................................Uuggacggagaacugauaagggc...................................	1	1	Fm1
.......................................................cuggacggagaCcugauaagggc...................................	1	1	Fm1
.......................................................cuggacggagaacugauaagggA...................................	37	1	Fm1
.......................................................cuggacggagaacugauUagggc...................................	1	1	Fm1
.......................................................Nuggacggagaacugauaagggc...................................	1	1	Fm1
.......................................................cuggacggagaacugauaGgggc...................................	9	1	Fm1
.......................................................cuggacggagaacugauaagggc...................................	422	0	Fm1
.......................................................cuggacggagaacugauaagggAu..................................	5	1	Fm1
.......................................................cuggacggagaacugauaagggcu..................................	14	0	Fm1
.......................................................cuggacggagaacugauaagggcG..................................	1	1	Fm1
.......................................................cuggacggagaacuAauaagggcu..................................	1	1	Fm1
........................................................uggacggagaacugauCa.......................................	3	1	Fm1
........................................................uggacggagaacugauaG.......................................	14	1	Fm1
........................................................uggacggagaacugUuaa.......................................	1	1	Fm1
........................................................uggacggagaacugCuaa.......................................	1	1	Fm1
........................................................uggacggUgaacugauaa.......................................	2	1	Fm1
........................................................Nggacggagaacugauaa.......................................	1	1	Fm1
........................................................uggacggagaacugauGa.......................................	1	1	Fm1
........................................................uggacggagaacugauUa.......................................	9	1	Fm1
........................................................ugAacggagaacugauaa.......................................	1	1	Fm1
........................................................uggacggagaacugauaU.......................................	88	1	Fm1
........................................................uggGcggagaacugauaa.......................................	1	1	Fm1
........................................................uggacggagaacugaAaa.......................................	215	1	Fm1
........................................................uggacggagUacugauaa.......................................	1	1	Fm1
........................................................uggacggagaacugaGaa.......................................	6	1	Fm1
........................................................uggacggagaCcugauaa.......................................	1	1	Fm1
........................................................uggacggagaGcugauaa.......................................	10	1	Fm1
```

Star **Mature**

gaauuuguccgaaagcggguccucgucauuuuuccgggccuguuuuuauacccuacuggacggagaacugauaagggcucgaauaggaccaagcaacauuugacguuaucugu			
....uAgacggagaacugauaa.......................................	2	1	Fm1
....uggacggagaacugauaC.......................................	15	1	Fm1
....uggacggagaacugauaa.......................................	2454	0	Fm1
....uggacNgagaacugauaag......................................	4	1	Fm1
....uggacggagaacugauUag......................................	24	1	Fm1
....uggacggaUaacugauaag......................................	4	1	Fm1
....uggacgCagaacugauaag......................................	1	1	Fm1
....uggacggagaacugauCag......................................	39	1	Fm1
....uggacggagaacugauaaU......................................	4300	1	Fm1
....Nggacggagaacugauaag......................................	10	1	Fm1
....uggacggagaacugauaCg......................................	70	1	Fm1
....uggacAgagaacugauaag......................................	5	1	Fm1
....uggacggagaaAugauaag......................................	1	1	Fm1
....uggUcggagaacugauaag......................................	5	1	Fm1
....uggaAggagaacugauaag......................................	1	1	Fm1
....ugUacggagaacugauaag......................................	1	1	Fm1
....uggacgUagaacugauaag......................................	5	1	Fm1
....uggGcggagaacugauaag......................................	8	1	Fm1
....Cggacggagaacugauaag......................................	2	1	Fm1
....uggacggagaacugUuaag......................................	6	1	Fm1
....uggacggagaacugauGag......................................	21	1	Fm1
....uggaGggagaacugauaag......................................	1	1	Fm1
....uAgacggagaacugauaag......................................	10	1	Fm1
....uggacggagaacugauaGg......................................	72	1	Fm1
....uggacggagaacugauaUg......................................	63	1	Fm1
....uggacggagaacCgauaag......................................	4	1	Fm1
....uggacggagaacugauaaC......................................	30	1	Fm1
....Aggacggagaacugauaag......................................	3	1	Fm1
....uggacggagaCcugauaag......................................	24	1	Fm1
....uggacggagaacugCuaag......................................	1	1	Fm1
....uggacggagaacuUauaag......................................	3	1	Fm1
....uggacggagaacGgauaag......................................	1	1	Fm1
....uggacggagUacugauaag......................................	3	1	Fm1
....uUgacggagaacugauaag......................................	1	1	Fm1
....uggacggagaacuCauaag......................................	2	1	Fm1
....uNgacggagaacugauaag......................................	1	1	Fm1
....uggacggagaacugauaag......................................	17744	0	Fm1
....ugAacggagaacugauaag......................................	5	1	Fm1
....uggacggagaaUugauaag......................................	8	1	Fm1
....uggacggaAaacugauaag......................................	3	1	Fm1
....uggacggUgaacugauaag......................................	4	1	Fm1
....uggacCgagaacugauaag......................................	1	1	Fm1
....uggacggagCacugauaag......................................	2	1	Fm1
....uggacUgagaacugauaag......................................	5	1	Fm1
....uggacggagaGcugauaag......................................	31	1	Fm1
....uggacggGgaacugauaag......................................	4	1	Fm1
....ugCacggagaacugauaag......................................	2	1	Fm1
....uggacggagaacugaAaag......................................	19	1	Fm1
....uggacgAagaacugauaag......................................	3	1	Fm1
....uggacggagaacugaCaag......................................	2	1	Fm1
....uggacggagaacuAauaag......................................	2	1	Fm1
....uggacggagaacugauaaA......................................	6927	1	Fm1
....uggaUggagaacugauaag......................................	4	1	Fm1
....uggacggagaacAgauaag......................................	1	1	Fm1
....uggCcggagaacugauaag......................................	1	1	Fm1
....uggacggagGacugauaag......................................	9	1	Fm1
....uggacggagaacAgauaagg.....................................	2	1	Fm1
....uggacCgagaacugauaagg.....................................	2	1	Fm1
....uggacggagaCcugauaagg.....................................	71	1	Fm1
....uggacggagaacCgauaagg.....................................	3	1	Fm1
....uggacggagaaUugauaagg.....................................	8	1	Fm1
....uggacggagUacugauaagg.....................................	17	1	Fm1
....ugUacggagaacugauaagg.....................................	5	1	Fm1
....uggacNgagaacugauaagg.....................................	2	1	Fm1
....uggacggagaacugauUagg.....................................	70	1	Fm1
....uggacAgagaacugauaagg.....................................	5	1	Fm1
....ugCacggagaacugauaagg.....................................	1	1	Fm1
....uggacggagaacugauaCgg.....................................	177	1	Fm1
....Cggacggagaacugauaagg.....................................	9	1	Fm1
....Aggacggagaacugauaagg.....................................	17	1	Fm1

```
                    Star                         Mature
gaauuuguccgaaagcggguccucgucauuuuuccgggccuguuuuuauacccuacuggacggagaacugauaagggcucgaauaggaccaagcaacauuugacguuaucugu
........................................................uggacggCgaacugauaagg.....................................  4      1  Fm1
........................................................uggGcggagaacugauaagg.....................................  16     1  Fm1
........................................................uggacggagaacugauaagg.....................................  46362  0  Fm1
........................................................uggacggagaacugauCagg.....................................  122    1  Fm1
........................................................uggacgAagaacugauaagg.....................................  4      1  Fm1
........................................................uggacggUgaacugauaagg.....................................  18     1  Fm1
........................................................uggacggagaacuUauaagg.....................................  1      1  Fm1
........................................................Nggacggagaacugauaagg.....................................  17     1  Fm1
........................................................uUgacggagaacugauaagg.....................................  8      1  Fm1
........................................................uggacggaUaacugauaagg.....................................  8      1  Fm1
........................................................uggacggagaacugauaaUg.....................................  3      1  Fm1
........................................................uggaUggagaacugauaagg.....................................  9      1  Fm1
........................................................uggacggaAaacugauaagg.....................................  2      1  Fm1
........................................................uggacggagaacuAauaagg.....................................  9      1  Fm1
........................................................uggacgCagaacugauaagg.....................................  2      1  Fm1
........................................................uggacggagaGcugauaagg.....................................  149    1  Fm1
........................................................uggacggagaaGugauaagg.....................................  1      1  Fm1
........................................................uggacggagCacugauaagg.....................................  6      1  Fm1
........................................................uAgacggagaacugauaagg.....................................  27     1  Fm1
........................................................uggacggGgaacugauaagg.....................................  8      1  Fm1
........................................................uggCcggagaacugauaagg.....................................  9      1  Fm1
........................................................ugAacggagaacugauaagg.....................................  9      1  Fm1
........................................................uggacggagaacugUuaagg.....................................  9      1  Fm1
........................................................uggacggagaacugauaagC.....................................  31     1  Fm1
........................................................uggacggagaacugGuaagg.....................................  7      1  Fm1
........................................................uggacggagaacugauaaAg.....................................  266    1  Fm1
........................................................uggaAggagaacugauaagg.....................................  1      1  Fm1
........................................................uggacggagaacuCauaagg.....................................  4      1  Fm1
........................................................uggacggagaacugauaagA.....................................  4462   1  Fm1
........................................................uNgacggagaacugauaagg.....................................  2      1  Fm1
........................................................uggacggagaacugauaaCg.....................................  4      1  Fm1
........................................................uggacggagaacugauaGgg.....................................  199    1  Fm1
........................................................uggacggagaacugaAaagg.....................................  2      1  Fm1
........................................................uggacggagaacugCuaagg.....................................  2      1  Fm1
........................................................uggacgUagaacugauaagg.....................................  8      1  Fm1
........................................................uggacggagGacugauaagg.....................................  51     1  Fm1
........................................................uggacggagaacugauaagU.....................................  1110   1  Fm1
........................................................uggacggaCaacugauaagg.....................................  1      1  Fm1
........................................................uggacUgagaacugauaagg.....................................  14     1  Fm1
........................................................uggUcggagaacugauaagg.....................................  8      1  Fm1
........................................................uggacggagaacugauaUgg.....................................  165    1  Fm1
........................................................uggacggagaacugaCaagg.....................................  2      1  Fm1
........................................................uggacgNagaacugauaagg.....................................  2      1  Fm1
........................................................uggacggagaacugauGagg.....................................  63     1  Fm1
........................................................uCgacggagaacugauaagg.....................................  4      1  Fm1
........................................................uggacggagaacugauaagCg....................................  5      1  Fm1
........................................................uggacggagaacAgauaaggg....................................  4      1  Fm1
........................................................uggacggagaacugauaUggg....................................  223    1  Fm1
........................................................uggacggaUaacugauaaggg....................................  14     1  Fm1
........................................................uggacggagaacugUuaaggg....................................  11     1  Fm1
........................................................uggUcggagaacugauaaggg....................................  6      1  Fm1
........................................................uggacggagaacugauCaggg....................................  154    1  Fm1
........................................................uggacgNagaacugauaaggg....................................  5      1  Fm1
........................................................uAgacggagaacugauaaggg....................................  30     1  Fm1
........................................................ugCacggagaacugauaaggg....................................  1      1  Fm1
........................................................uggacggagaacCgauaaggg....................................  12     1  Fm1
........................................................Gggacggagaacugauaaggg....................................  1      1  Fm1
........................................................uggacggagaacugauaaggU....................................  1894   1  Fm1
........................................................uCgacggagaacugauaaggg....................................  5      1  Fm1
........................................................uggacggGgaacugauaaggg....................................  18     1  Fm1
........................................................uggacggagaacugauaaggC....................................  348    1  Fm1
........................................................uggacggagaacugauaaggg....................................  58344  0  Fm1
........................................................uggacggagaacugauGaggg....................................  97     1  Fm1
........................................................uggacggagaacugauaGggg....................................  524    1  Fm1
........................................................uggaAggagaacugauaaggg....................................  1      1  Fm1
........................................................ugUacggagaacugauaaggg....................................  3      1  Fm1
........................................................uggacggagaacugauaagAg....................................  22     1  Fm1
........................................................uggacggagaacuAauaaggg....................................  13     1  Fm1
........................................................uggacggaAaacugauaaggg....................................  2      1  Fm1
........................................................uggacgUagaacugauaaggg....................................  16     1  Fm1
```

```
                    Star                               Mature
gaauuuguccgaaagcggguccucgucauuuuuccgggccuguuuuuauacccuacuggacggagaacugauaagggcucgaauaggaccaagcaacauuugacguuaucugu
.......................................................uggacggagaacugCuaaggg.....................................    4      1    Fm1
.......................................................uggacggagaGcugauaaggg.....................................    136    1    Fm1
.......................................................uggacgCagaacugauaaggg.....................................    3      1    Fm1
.......................................................uggacggagaacugauaaagUg.....................................    14     1    Fm1
.......................................................uggacggagaacuUauaaggg.....................................    4      1    Fm1
.......................................................uggacgAagaacugauaaggg.....................................    8      1    Fm1
.......................................................uggaGggagaacugauaaggg.....................................    3      1    Fm1
.......................................................uggacggagaacugaGaaggg.....................................    2      1    Fm1
.......................................................uggacggagaacugGuaaggg.....................................    13     1    Fm1
.......................................................uggacggagaacugauaCggg.....................................    225    1    Fm1
.......................................................ugAacggagaacugauaaggg.....................................    12     1    Fm1
.......................................................uggacggagaacugauaaAgg.....................................    6      1    Fm1
.......................................................uggacggagGacugauaaggg.....................................    59     1    Fm1
.......................................................uggacggUgaacugauaaggg.....................................    20     1    Fm1
.......................................................Nggacggagaacugauaaggg.....................................    26     1    Fm1
.......................................................uggGcggagaacugauaaggg.....................................    14     1    Fm1
.......................................................uggacggagaaUugauaaggg.....................................    20     1    Fm1
.......................................................uggacggagaacugaCaaggg.....................................    2      1    Fm1
.......................................................uggacggagUacugauaaggg.....................................    19     1    Fm1
.......................................................uggCcggagaacugauaaggg.....................................    10     1    Fm1
.......................................................uggacggagaacugauaaUgg.....................................    8      1    Fm1
.......................................................Cggacggagaacugauaaggg.....................................    9      1    Fm1
.......................................................uggacggagaacugauUaggg.....................................    93     1    Fm1
.......................................................ugNacggagaacugauaaggg.....................................    1      1    Fm1
.......................................................uggacNgagaacugauaaggg.....................................    11     1    Fm1
.......................................................uggacggagaacugaAaaggg.....................................    2      1    Fm1
.......................................................uggacAgagaacugauaaggg.....................................    8      1    Fm1
.......................................................uggaUggagaacugauaaggg.....................................    13     1    Fm1
.......................................................uggacggagaCcugauaaggg.....................................    72     1    Fm1
.......................................................uggacUgagaacugauaaggg.....................................    15     1    Fm1
.......................................................uggacggaCaacugauaaggg.....................................    3      1    Fm1
.......................................................uggacggagaacugauaaggA.....................................    11343  1    Fm1
.......................................................uggacCgagaacugauaaggg.....................................    4      1    Fm1
.......................................................Aggacggagaacugauaaggg.....................................    24     1    Fm1
.......................................................uggacggCgaacugauaaggg.....................................    5      1    Fm1
.......................................................uUgacggagaacugauaaggg.....................................    14     1    Fm1
.......................................................uggacggagaacuCauaaggg.....................................    5      1    Fm1
.......................................................uggacggagaacGgauaaggg.....................................    1      1    Fm1
.......................................................uggacggagCacugauaaggg.....................................    8      1    Fm1
.......................................................uggacggagaacugauUagggc....................................    1143   1    Fm1
.......................................................uggUcggagaacugauaagggc....................................    135    1    Fm1
.......................................................ugUacggagaacugauaagggc....................................    131    1    Fm1
.......................................................uggacggagaacugauGagggc....................................    1216   1    Fm1
.......................................................uggacggagUacugauaagggc....................................    282    1    Fm1
.......................................................uggacggagaacuAauaagggc....................................    170    1    Fm1
.......................................................uggacggagaacugauaaagAgc....................................    127    1    Fm1
.......................................................uggacggagaacugaGaagggc....................................    21     1    Fm1
.......................................................uggacggagaacugCuaagggc....................................    25     1    Fm1
.......................................................uggacgUagaacugauaagggc....................................    178    1    Fm1
.......................................................ugCacggagaacugauaagggc....................................    65     1    Fm1
.......................................................uggacggagaGcugauaagggc....................................    1253   1    Fm1
.......................................................uggacggagaaUugauaagggc....................................    284    1    Fm1
.......................................................uggacggaCaacugauaagggc....................................    44     1    Fm1
.......................................................uggacggagaacugauaGgggc....................................    8811   1    Fm1
.......................................................uggCcggagaacugauaagggc....................................    174    1    Fm1
.......................................................uggacggagaacugauaaggCc....................................    116    1    Fm1
.......................................................uggacggagaacugauaaUggc....................................    128    1    Fm1
.......................................................Cggacggagaacugauaagggc....................................    102    1    Fm1
.......................................................uAgacggagaacugauaagggc....................................    390    1    Fm1
.......................................................uggacgAagaacugauaagggc....................................    98     1    Fm1
.......................................................uggacggagaacGgauaagggc....................................    33     1    Fm1
.......................................................uggacUgagaacugauaagggc....................................    145    1    Fm1
.......................................................uNgacggagaacugauaagggc....................................    7      1    Fm1
.......................................................uggacggagaacugauaagggG....................................    874    1    Fm1
.......................................................uggacggagaacAgauaagggc....................................    31     1    Fm1
.......................................................uUgacggagaacugauaagggc....................................    193    1    Fm1
.......................................................uggacggagaacuUauaagggc....................................    78     1    Fm1
.......................................................uggacgNagaacugauaagggc....................................    46     1    Fm1
.......................................................uggacCgagaacugauaagggc....................................    34     1    Fm1
.......................................................uggaAggagaacugauaagggc....................................    66     1    Fm1
```

```
                    Star                                  Mature
gaauuuguccgaaagcggguccucgucauuuuuccgggccuguuuuuauacccuacuggacggagaacugauaagggcucgaauaggaccaagcaacauuugacguuaucugu
........................................................uggacggagaacugGuaagggc...................................   99       1    Fm1
........................................................uggacggaUaacugauaagggc...................................   154      1    Fm1
........................................................uggacggagaaGugauaagggc...................................   18       1    Fm1
........................................................uggacggagaacugauaNgggc...................................   2        1    Fm1
........................................................Nggacggagaacugauaagggc...................................   440      1    Fm1
........................................................uggacggagaacuCauaagggc...................................   49       1    Fm1
........................................................uggacggagaaAugauaagggc...................................   22       1    Fm1
........................................................uCgacggagaacugauaagggc...................................   73       1    Fm1
........................................................uggacggagaacugauaaCggc...................................   16       1    Fm1
........................................................Aggacggagaacugauaagggc...................................   153      1    Fm1
........................................................uggacggagaCcugauaagggc...................................   672      1    Fm1
........................................................uggacggagaacugaAaagggc...................................   18       1    Fm1
........................................................uggacggagaacugUuaagggc...................................   110      1    Fm1
........................................................uggacgCagaacugauaagggc...................................   47       1    Fm1
........................................................uggacAgagaacugauaagggc...................................   137      1    Fm1
........................................................ugAacggagaacugauaagggc...................................   183      1    Fm1
........................................................Gggacggagaacugauaagggc...................................   22       1    Fm1
........................................................uggacggagaacugauaagggc...................................   846342   0    Fm1
........................................................uggacggagGacugauaagggc...................................   1684     1    Fm1
........................................................uggacggGgaacugauaagggc...................................   194      1    Fm1
........................................................uggacggagaacugauCagggc...................................   1755     1    Fm1
........................................................uggacggCgaacugauaagggc...................................   86       1    Fm1
........................................................uggacggagCacugauaagggc...................................   169      1    Fm1
........................................................uggacggagaacugauaUgggc...................................   2648     1    Fm1
........................................................uggacggagaacugauaaggAc...................................   313      1    Fm1
........................................................uggacggagaacCgauaagggc...................................   121      1    Fm1
........................................................uggaUggagaacugauaagggc...................................   194      1    Fm1
........................................................uggacggagaacugauaaAggc...................................   61       1    Fm1
........................................................uggacggUgaacugauaagggc...................................   431      1    Fm1
........................................................uggacggagaacugauaagUgc...................................   175      1    Fm1
........................................................uggacggagaacugauaCgggc...................................   3205     1    Fm1
........................................................uggacNgagaacugauaagggc...................................   135      1    Fm1
........................................................uggaGggagaacugauaagggc...................................   38       1    Fm1
........................................................uggGcggagaacugauaagggc...................................   170      1    Fm1
........................................................ugNacggagaacugauaagggc...................................   4        1    Fm1
........................................................uggacggagaacugauaagCgc...................................   50       1    Fm1
........................................................uggacggagaacugauaaggUc...................................   223      1    Fm1
........................................................uggacggaAaacugauaagggc...................................   49       1    Fm1
........................................................uggacggagaacugaCaagggc...................................   80       1    Fm1
........................................................uggacggagaacugauaagggU...................................   7010     1    Fm1
........................................................uggacggagaacugauaagggA...................................   21574    1    Fm1
........................................................uggacggagaCcugauaagggcu..................................   32       1    Fm1
........................................................uggacggagaGcugauaagggcu..................................   58       1    Fm1
........................................................uggacggagaacugauaCgggcu..................................   143      1    Fm1
........................................................uggacggagaacugaAaagggcu..................................   1        1    Fm1
........................................................uggacggagaacugauCagggcu..................................   98       1    Fm1
........................................................uggCcggagaacugauaagggcu..................................   14       1    Fm1
........................................................uggacggagaacugauaaggUcu..................................   10       1    Fm1
........................................................uggGcggagaacugauaagggcu..................................   8        1    Fm1
........................................................uggacgAagaacugauaagggcu..................................   5        1    Fm1
........................................................uggaGggagaacugauaagggcu..................................   3        1    Fm1
........................................................uggacggagaacugauaagggUu..................................   885      1    Fm1
........................................................uggacgUagaacugauaagggcu..................................   10       1    Fm1
........................................................uggUcggagaacugauaagggcu..................................   9        1    Fm1
........................................................ugCacggagaacugauaagggcu..................................   3        1    Fm1
........................................................uggacggagaacAgauaagggcu..................................   1        1    Fm1
........................................................uggacAgagaacugauaagggcu..................................   2        1    Fm1
........................................................uggacggagaacugauaaNggcu..................................   1        1    Fm1
........................................................uggacggagaacugaCaagggcu..................................   5        1    Fm1
........................................................uggaAggagaacugauaagggcu..................................   6        1    Fm1
........................................................uggacggagaacugaGaagggcu..................................   1        1    Fm1
........................................................uggacggagaacugauGagggcu..................................   61       1    Fm1
........................................................uggacggagaacugauaaUggcu..................................   6        1    Fm1
........................................................uggacggagaaAugauaagggcu..................................   2        1    Fm1
........................................................uggacggagaacuAauaagggcu..................................   9        1    Fm1
........................................................uggacggagaacugauaUgggcu..................................   138      1    Fm1
........................................................uCgacggagaacugauaagggcu..................................   6        1    Fm1
........................................................uggacggagUacugauaagggcu..................................   12       1    Fm1
........................................................uggacggagaacugauaGgggcu..................................   520      1    Fm1
........................................................uggaUggagaacugauaagggcu..................................   7        1    Fm1
```

```
                    Star                               Mature
gaauuuguccgaaagcggguccucgucauuuuuccgggccuguuuuuauacccuacuggacggagaacugauaagggcucgaauaggaccaagcaacauuugacguuaucugu
........................................................uggacggagaacCgauaagggcu..................................    8      1    Fm1
........................................................uggacNgagaacugauaagggcu..................................    6      1    Fm1
........................................................Nggacggagaacugauaagggcu..................................    14     1    Fm1
........................................................uggacggagaacuCauaagggcu..................................    2      1    Fm1
........................................................ugAacggagaacugauaagggcu..................................    3      1    Fm1
........................................................uggacggagaacugauaagggcu..................................    40145  0    Fm1
........................................................uggacggagGacugauaagggcu..................................    74     1    Fm1
........................................................Aggacggagaacugauaagggcu..................................    4      1    Fm1
........................................................uggacggagaacugauaagggGu..................................    13     1    Fm1
........................................................uggacggagaacugUuaagggcu..................................    7      1    Fm1
........................................................uggacggagaacugauaagggcC..................................    616    1    Fm1
........................................................uggacggUgaacugauaagggcu..................................    16     1    Fm1
........................................................uggacggagaacugGuaagggcu..................................    3      1    Fm1
........................................................uggacggagaaUugauaagggcu..................................    12     1    Fm1
........................................................ugNacggagaacugauaagggcu..................................    2      1    Fm1
........................................................uggacUgagaacugauaagggcu..................................    5      1    Fm1
........................................................uUgacggagaacugauaagggcu..................................    10     1    Fm1
........................................................uggacgNagaacugauaagggcu..................................    3      1    Fm1
........................................................uggacggagaacugauaagggAu..................................    1469   1    Fm1
........................................................Cggacggagaacugauaagggcu..................................    5      1    Fm1
........................................................uggacggagaacugauUagggcu..................................    56     1    Fm1
........................................................uggacggaAaacugauaagggcu..................................    2      1    Fm1
........................................................uggacggagaacugauaaggCcu..................................    4      1    Fm1
........................................................ugUacggagaacugauaagggcu..................................    9      1    Fm1
........................................................uggacggagaacugCuaagggcu..................................    1      1    Fm1
........................................................uggacggaUaacugauaagggcu..................................    5      1    Fm1
........................................................uggacggGgaacugauaagggcu..................................    9      1    Fm1
........................................................uggacgCagaacugauaagggcu..................................    4      1    Fm1
........................................................uggacggagaaGugauaagggcu..................................    1      1    Fm1
........................................................uggacggagCacugauaagggcu..................................    14     1    Fm1
........................................................uggacggCgaacugauaagggcu..................................    4      1    Fm1
........................................................uggacggagaacugauaagAgcu..................................    7      1    Fm1
........................................................uggacggagaacugauaagUgcu..................................    5      1    Fm1
........................................................uggacggagaacugauaaAggcu..................................    3      1    Fm1
........................................................uggacggaCaacugauaagggcu..................................    5      1    Fm1
........................................................uggacggagaacuUauaagggcu..................................    5      1    Fm1
........................................................uggacggagaacugauaaggAcu..................................    5      1    Fm1
........................................................uAgacggagaacugauaagggcu..................................    21     1    Fm1
........................................................uggacggagaacugauaagCgcu..................................    3      1    Fm1
........................................................uggacggagaacugauaagggcG..................................    2275   1    Fm1
........................................................uggacggUgaacugauaagggcuc.................................    1      1    Fm1
........................................................uggacggagaacugauaagggcAc.................................    138    1    Fm1
........................................................uggacggagaUcugauaagggcuc.................................    10     1    Fm1
........................................................uggacggagaacugauaagggcuc.................................    203    0    Fm1
........................................................uggacggagaacugauaagggcCc.................................    5      1    Fm1
........................................................uggacggagaacugauaagggcuA.................................    217    1    Fm1
........................................................uggacggagaacugauaagggAuc.................................    6      1    Fm1
........................................................uggacggagaacugauaCgggcuc.................................    1      1    Fm1
........................................................uggacggagaacugauaagggUuc.................................    2      1    Fm1
........................................................uggacggagaacugauaagggcuG.................................    63     1    Fm1
........................................................uggacggagaacugauaagggcucU................................    28     1    Fm1
........................................................uggacggagaacugauaagggcAcg................................    1      1    Fm1
........................................................uggacggagaacugauaagggcucA................................    1      1    Fm1
........................................................uggacggagaacugauaagggcuGgaa..............................    1      1    Fm1
.........................................................ggacggagaacugauaUg......................................    1      1    Fm1
.........................................................ggacggagaacugauaag......................................    21     0    Fm1
.........................................................ggGcggagaacugauaag......................................    1      1    Fm1
.........................................................ggacggagaacugauaaU......................................    16     1    Fm1
.........................................................Ugacggagaacugauaag......................................    1      1    Fm1
.........................................................ggacggaAaacugauaag......................................    1      1    Fm1
.........................................................ggacggagaacugauaaA......................................    8      1    Fm1
.........................................................ggacggagGacugauaag......................................    1      1    Fm1
.........................................................ggacggagaacugauaagg.....................................    60     0    Fm1
.........................................................ggacggagaacugauaagA.....................................    4      1    Fm1
.........................................................Ugacggagaacugauaagg.....................................    2      1    Fm1
.........................................................ggacggagaacugauaagU.....................................    1      1    Fm1
.........................................................ggacggagaacGgauaagg.....................................    1      1    Fm1
.........................................................ggacggagaaAugauaagg.....................................    1      1    Fm1
.........................................................ggacggagaacugauaaggg....................................    73     0    Fm1
.........................................................ggacggagaacugauaaggA....................................    16     1    Fm1
```

Star **Mature**

```
gaauuuguccgaaagcgggucucgucauuuuuccgggccuguuuuuauacccuacuggacggagaacugauaagggcucgaauaggaccaagcaacauuugacguuaucugu
.........................................................ggacggagaaAugauaaggg....................................   1     1   Fm1
.........................................................ggacggagaacugauaaggC....................................   1     1   Fm1
.........................................................ggacCgagaacugauaaggg....................................   1     1   Fm1
.........................................................ggacggagCacugauaaggg....................................   1     1   Fm1
.........................................................ggacggagaacugauCaggg....................................   1     1   Fm1
.........................................................ggacggagaacugauGaggg....................................   1     1   Fm1
.........................................................Ugacggagaacugauaaggg....................................   4     1   Fm1
.........................................................ggacggagaacugauaaggU....................................   4     1   Fm1
.........................................................ggacggagaacugauaagUgc...................................   2     1   Fm1
.........................................................ggacgUagaacugauaagggc...................................   1     1   Fm1
.........................................................ggacggUgaacugauaagggc...................................   1     1   Fm1
.........................................................ggacggagaacugauaaggAc...................................   1     1   Fm1
.........................................................ggacggagaacCgauaagggc...................................   3     1   Fm1
.........................................................ggacggagaacugauaGgggc...................................   9     1   Fm1
.........................................................ggacggagaacugauaaggUc...................................   2     1   Fm1
.........................................................ggacggCgaacugauaagggc...................................   4     1   Fm1
.........................................................ggacggagaaUugauaagggc...................................   4     1   Fm1
.........................................................ggaGggagaacugauaagggc...................................   1     1   Fm1
.........................................................ggacggagaacAgauaagggc...................................   1     1   Fm1
.........................................................ggacggagaacugauaagAgc...................................   2     1   Fm1
.........................................................ggacggagCacugauaagggc...................................   16    1   Fm1
.........................................................ggacggGgaacugauaagggc...................................   1     1   Fm1
.........................................................ggacggagGacugauaagggc...................................   4     1   Fm1
.........................................................ggacggagaacugauaagggc...................................   1101  0   Fm1
.........................................................ggacggagaacugauaUgggc...................................   1     1   Fm1
.........................................................ggCcggagaacugauaagggc...................................   2     1   Fm1
.........................................................ggacggagaCcugauaagggc...................................   3     1   Fm1
.........................................................ggacggagaaAugauaagggc...................................   2     1   Fm1
.........................................................ggacggagUacugauaagggc...................................   2     1   Fm1
.........................................................ggacggaUaacugauaagggc...................................   1     1   Fm1
.........................................................ggacggagaaGugauaagggc...................................   1     1   Fm1
.........................................................ggacggagaacugauaagggU...................................   12    1   Fm1
.........................................................ggacUgagaacugauaagggc...................................   1     1   Fm1
.........................................................ggGcggagaacugauaagggc...................................   2     1   Fm1
.........................................................ggaAggagaacugauaagggc...................................   4     1   Fm1
.........................................................Ugacggagaacugauaagggc...................................   45    1   Fm1
.........................................................ggacggagaacugauCagggc...................................   1     1   Fm1
.........................................................gUacggagaacugauaagggc...................................   1     1   Fm1
.........................................................ggacggaAaacugauaagggc...................................   2     1   Fm1
.........................................................ggacggagaacGgauaagggc...................................   1     1   Fm1
.........................................................ggacggagaacugUuaagggc...................................   1     1   Fm1
.........................................................ggacggagaacugauaaggCc...................................   4     1   Fm1
.........................................................ggacggagaacugaAaagggc...................................   2     1   Fm1
.........................................................ggacggagaacugauUagggc...................................   1     1   Fm1
.........................................................ggacggagaacugauaCgggc...................................   1     1   Fm1
.........................................................ggacggagaacugauaagggA...................................   51    1   Fm1
.........................................................Ugacggagaacugauaagggcu..................................   1     1   Fm1
.........................................................ggacggagaacugauaagggcG..................................   4     1   Fm1
.........................................................ggacggagaacugauaagggcu..................................   48    0   Fm1
.........................................................ggacggagaacugauaagggAu..................................   2     1   Fm1
.........................................................ggacggagaacugauaagggcAc.................................   1     1   Fm1
..........................................................Uacggagaacugauaagggc...................................   3     1   Fm1
..........................................................gacggagaacugauaagggc...................................   4     0   Fm1
............................................................cggagaacugauaagggc...................................   3     0   Fm1
............................................................Uggagaacugauaagggc...................................   10    1   Fm1

....................ccucgucauuuuuUcgggccugu......................................................................   1     1   Fm2
....................ccucgucauuuuuccggAccugu......................................................................   1     1   Fm2
....................Ncucgucauuuuuccgggccugu......................................................................   1     1   Fm2
....................ccucgucauuuuuccggUccugu......................................................................   1     1   Fm2
....................Ucucgucauuuuuccgggccugu......................................................................   5     1   Fm2
....................ccucgucauuuuuccgggccugG......................................................................   5     1   Fm2
....................ccucgucauuuuuccgggccugu......................................................................   212   0   Fm2
....................ccucgucauuAuuccgggccugu......................................................................   1     1   Fm2
....................ccucgucauuuuuccgggccuguu.....................................................................   55    0   Fm2
....................Ucucgucauuuuuccgggccuguu.....................................................................   1     1   Fm2
....................ccucgucauuuuuccgggccuguG.....................................................................   1     1   Fm2
....................ccucgucauuuuuccggUccuguuu....................................................................   1     1   Fm2
....................ccucgucauuuuuccgggccuguuu....................................................................   39    0   Fm2
....................ccucgucauuuuuccgggccugGuuu...................................................................   1     1   Fm2
```

```
                    Star                               Mature
gaauuuguccgaaagcggguccucgucauuuuuccgggccuguuuuuauacccuacuggacggagaacugauaagggcucgaauaggaccaagcaacauuugacguuaucugu
....................ccucgucauuuuuccgggccuguuuu...................................................................    19    0    Fm2
....................ccucgucauuAuccgggccuguuuu....................................................................    1     1    Fm2
....................ccucgucauuuuuccgggccuguuuuuauacccuacu........................................................    5     0    Fm2
.....................cucgucauuuuuccgggAcugu......................................................................    1     1    Fm2
.....................cucgucauuuuuccgggccugu......................................................................    12    0    Fm2
.....................cucgucauuuuuccgggccuguu.....................................................................    18    0    Fm2
...........................................uuuuauacccuacuggacggagaac.............................................    21    0    Fm2
.....................................................uacuggacggagaacugauaagggc...................................    11    0    Fm2
......................................................acuggacggagaacugaGaagg.....................................    1     1    Fm2
......................................................acuggacggagaacugauaagg.....................................    13    0    Fm2
.......................................................cuggacggagaacugauU........................................    2     1    Fm2
.......................................................cuggacggagaacugaua........................................    9     0    Fm2
.......................................................cuggacggagaacugauaa.......................................    17    0    Fm2
.......................................................cuggacggagaacugauaag......................................    51    0    Fm2
.......................................................cuggacggagaacugauCag......................................    1     1    Fm2
.......................................................cuggacggagaacugaCaag......................................    2     1    Fm2
.......................................................cuggacggagaacugauaaU......................................    11    1    Fm2
.......................................................cuggacggagaacugauaaA......................................    3     1    Fm2
.......................................................cuggacggagaacugauaaAg.....................................    6     1    Fm2
.......................................................cuggacggagaacugaGaagg.....................................    4     1    Fm2
.......................................................cuggacggGgaacugauaagg.....................................    1     1    Fm2
.......................................................cuggacggagaacugauGagg.....................................    2     1    Fm2
.......................................................cuAgacggagaacugauaagg.....................................    1     1    Fm2
.......................................................cuggacggagCacugauaagg.....................................    1     1    Fm2
.......................................................cuggacggagGacugauaagg.....................................    1     1    Fm2
.......................................................cuggacggagaacugauaagA.....................................    6     1    Fm2
.......................................................cuggacggagaacugauaagU.....................................    3     1    Fm2
.......................................................cuggacggagaacugauaagg.....................................    228   0    Fm2
.......................................................Uuggacggagaacugauaaggg....................................    1     1    Fm2
.......................................................cuggacggagaacugauaaggA....................................    31    1    Fm2
.......................................................cuggacggagaacugaCaaggg....................................    1     1    Fm2
.......................................................cuggacggagaacugauaaggU....................................    45    1    Fm2
.......................................................cuggacggagaacugauCaggg....................................    2     1    Fm2
.......................................................cuggacggagaacugauaaggg....................................    210   0    Fm2
.......................................................cuggacggagaacugaGaaggg....................................    1     1    Fm2
.......................................................cuggacggagaacugauaagAg....................................    1     1    Fm2
.......................................................cuggacggagaacugauaaAgg....................................    7     1    Fm2
.......................................................cuggacggagaacugauaagggc...................................    420   0    Fm2
.......................................................cuggacggagaacugauaaUggc...................................    1     1    Fm2
.......................................................cuggacggagCacugauaagggc...................................    1     1    Fm2
.......................................................cuggacggagaacugaGaagggc...................................    2     1    Fm2
.......................................................cuggacggagaacugauaagggU...................................    23    1    Fm2
.......................................................cuggacggagaacugaAaagggc...................................    2     1    Fm2
.......................................................cuggacggagaacugauaagggG...................................    2     1    Fm2
.......................................................cuggacggagaacugaCaagggc...................................    1     1    Fm2
.......................................................cuggacggagaacCgauaagggc...................................    1     1    Fm2
.......................................................cuggaUggagaacugauaagggc...................................    2     1    Fm2
.......................................................cuggacggagaacugauUagggc...................................    1     1    Fm2
.......................................................cuggacggagaaUugauaagggc...................................    4     1    Fm2
.......................................................cuggacggagaacugauCagggc...................................    2     1    Fm2
.......................................................cuggacggagaacugauaagggcu..................................    6     0    Fm2
........................................................uggacggagaCcugauaa.......................................    2     1    Fm2
........................................................uggacggagaacugauUa.......................................    9     1    Fm2
........................................................uggacggagaacugauaG.......................................    12    1    Fm2
........................................................uggacggaUaacugauaa.......................................    1     1    Fm2
........................................................uggacggagaacugaGaa.......................................    2     1    Fm2
........................................................uggacggagaacugauaa.......................................    908   0    Fm2
........................................................uggaUggagaacugauaa.......................................    1     1    Fm2
........................................................uggacggagaacugauaU.......................................    23    1    Fm2
........................................................uggacggagaacugauaC.......................................    3     1    Fm2
........................................................uggacggagaacugauGa.......................................    3     1    Fm2
........................................................uggacggagaGcugauaa.......................................    1     1    Fm2
........................................................uggacggagaacugaAaa.......................................    81    1    Fm2
........................................................uggacggagaacugauCa.......................................    1     1    Fm2
........................................................uggacggagaacCgauaag......................................    1     1    Fm2
........................................................uAgacggagaacugauaag......................................    6     1    Fm2
........................................................uggacggagaCcugauaag......................................    8     1    Fm2
........................................................uggacggagaacugauaaU......................................    1662  1    Fm2
........................................................uggacNgagaacugauaag......................................    2     1    Fm2
........................................................uggacggaUaacugauaag......................................    1     1    Fm2
```

Star Mature

```
gaauuuguccgaaagcggguccucgucauuuuuccgggccuguuuuuauacccuacuggacggagaacugauaagggcucgaauaggaccaagcaacauuugacguuaucugu
........................................................uggacggagUacugauaag......................................   4      1    Fm2
........................................................uggacggagaacugauaaC......................................   7      1    Fm2
........................................................uggacUgagaacugauaag......................................   2      1    Fm2
........................................................uggacggagaacugauCag......................................   15     1    Fm2
........................................................Cggacggagaacugauaag......................................   1      1    Fm2
........................................................uggCcggagaacugauaag......................................   1      1    Fm2
........................................................uggacggagaacugauGag......................................   9      1    Fm2
........................................................uggaGggagaacugauaag......................................   2      1    Fm2
........................................................uggacggagaacugauaGg......................................   19     1    Fm2
........................................................uggUcggagaacugauaag......................................   1      1    Fm2
........................................................uggacggagGacugauaag......................................   9      1    Fm2
........................................................uggacggagaacugaCaag......................................   3      1    Fm2
........................................................uggacggGgaacugauaag......................................   3      1    Fm2
........................................................uggacggagaacugauaUg......................................   17     1    Fm2
........................................................uggacggagaacAgauaag......................................   1      1    Fm2
........................................................uggacggagaGcugauaag......................................   18     1    Fm2
........................................................ugCacggagaacugauaag......................................   1      1    Fm2
........................................................uggacggagaacugauaCg......................................   22     1    Fm2
........................................................Nggacggagaacugauaag......................................   3      1    Fm2
........................................................uggacggCgaacugauaag......................................   1      1    Fm2
........................................................uggacggagaacugauaag......................................   7644   0    Fm2
........................................................Aggacggagaacugauaag......................................   1      1    Fm2
........................................................uUgacggagaacugauaag......................................   1      1    Fm2
........................................................uggacggagCacugauaag......................................   2      1    Fm2
........................................................uggacggagaacugauaaA......................................   3153   1    Fm2
........................................................uggacggUgaacugauaag......................................   2      1    Fm2
........................................................uggacggagaaUugauaag......................................   2      1    Fm2
........................................................uggacggagaacugauUag......................................   9      1    Fm2
........................................................ugAacggagaacugauaag......................................   3      1    Fm2
........................................................uggacggagaacugaAaag......................................   5      1    Fm2
........................................................uggacgUagaacugauaag......................................   2      1    Fm2
........................................................uggacAgagaacugauaag......................................   1      1    Fm2
........................................................uggGcggagaacugauaag......................................   1      1    Fm2
........................................................uggacggagaacuCauaag......................................   2      1    Fm2
........................................................uggaUggagaacugauaag......................................   9      1    Fm2
........................................................Cggacggagaacugauaagg.....................................   6      1    Fm2
........................................................ugAacggagaacugauaagg.....................................   6      1    Fm2
........................................................uggacgUagaacugauaagg.....................................   1      1    Fm2
........................................................uggacggaAaacugauaagg.....................................   1      1    Fm2
........................................................uggacggagaacugauaUgg.....................................   70     1    Fm2
........................................................uggacggagaacugaGaagg.....................................   2      1    Fm2
........................................................uggacggagGacugauaagg.....................................   23     1    Fm2
........................................................uggacggagaacugaCaagg.....................................   1      1    Fm2
........................................................uUgacggagaacugauaagg.....................................   9      1    Fm2
........................................................uggacNgagaacugauaagg.....................................   1      1    Fm2
........................................................uggacggGgaacugauaagg.....................................   7      1    Fm2
........................................................uggacggaUaacugauaagg.....................................   3      1    Fm2
........................................................uggacggaCaacugauaagg.....................................   3      1    Fm2
........................................................uggacggagaacugauGagg.....................................   32     1    Fm2
........................................................uggacgAagaacugauaagg.....................................   3      1    Fm2
........................................................uggacUgagaacugauaagg.....................................   3      1    Fm2
........................................................uggacggagaacugauaagU.....................................   534    1    Fm2
........................................................uggacggagaacuUauaagg.....................................   5      1    Fm2
........................................................uggacggagaacugauaagA.....................................   1695   1    Fm2
........................................................uggacggagaacCgauaagg.....................................   8      1    Fm2
........................................................uggaAggagaacugauaagg.....................................   3      1    Fm2
........................................................uggacggagaacugauaagg.....................................   23066  0    Fm2
........................................................uggacggagaGcugauaagg.....................................   103    1    Fm2
........................................................uggacggagaacugUuaagg.....................................   4      1    Fm2
........................................................uggCcggagaacugauaagg.....................................   5      1    Fm2
........................................................uggacggagaacugauaaUg.....................................   4      1    Fm2
........................................................uggacggagaacugauUagg.....................................   28     1    Fm2
........................................................uggacggagaacugauaGgg.....................................   112    1    Fm2
........................................................uggacggagaacuAauaagg.....................................   4      1    Fm2
........................................................uggacgNagaacugauaagg.....................................   2      1    Fm2
........................................................ugCacggagaacugauaagg.....................................   3      1    Fm2
........................................................uggacggagaacugGuaagg.....................................   5      1    Fm2
........................................................uggacggagaacuCauaagg.....................................   2      1    Fm2
........................................................uggacggagCacugauaagg.....................................   6      1    Fm2
........................................................uggacggagaacugauaagC.....................................   3      1    Fm2
```

Star Mature

gaauuuguccgaaagcggguccucgucauuuuuccgggccuguuuuuauacccuacuggacggagaacugauaagggcucgaauaggaccaagcaacauuugacguuaucugu			
....uggaUggagaacugauaagg..	6	1	Fm2
....Nggacggagaacugauaagg..	11	1	Fm2
....uggacggagaacugauCagg..	47	1	Fm2
....ugUacggagaacugauaagg..	4	1	Fm2
....uggacggUgaacugauaagg..	12	1	Fm2
....uggGcggagaacugauaagg..	3	1	Fm2
....uggacggagUacugauaagg..	7	1	Fm2
....uggacggagaCcugauaagg..	68	1	Fm2
....uggacggagaacugauaCgg..	93	1	Fm2
....uggacgCagaacugauaagg..	2	1	Fm2
....uggacAgagaacugauaagg..	5	1	Fm2
....uggacggagaacugauaaAg..	124	1	Fm2
....uggacggagaacGgauaagg..	1	1	Fm2
....uggaGggagaacugauaagg..	1	1	Fm2
....uggacCgagaacugauaagg..	1	1	Fm2
....Aggacggagaacugauaagg..	16	1	Fm2
....uggacggCgaacugauaagg..	4	1	Fm2
....uAgacggagaacugauaagg..	10	1	Fm2
....uggacggagaaGugauaagg..	1	1	Fm2
....uggacggagaacugauaaCg..	1	1	Fm2
....uggUcggagaacugauaagg..	4	1	Fm2
....uggacggagaaUugauaagg..	4	1	Fm2
....uggacggagaacugaCaaggg...	6	1	Fm2
....uCgacggagaacugauaaggg...	3	1	Fm2
....uggacggagaacugauaCggg...	130	1	Fm2
....uggaUggagaacugauaaggg...	8	1	Fm2
....uggacggUgaacugauaaggg...	21	1	Fm2
....uggacggagaacugUuaaggg...	6	1	Fm2
....Cggacggagaacugauaaggg...	8	1	Fm2
....ugCacggagaacugauaaggg...	4	1	Fm2
....uUgacggagaacugauaaggg...	10	1	Fm2
....uggacAgagaacugauaaggg...	7	1	Fm2
....uggacgNagaacugauaaggg...	4	1	Fm2
....uggacgAagaacugauaaggg...	2	1	Fm2
....uggacggagaacugauaaggg...	35348	0	Fm2
....uggacNgagaacugauaaggg...	4	1	Fm2
....uggacggagaacugauCaggg...	75	1	Fm2
....uggacggCgaacugauaaggg...	2	1	Fm2
....uggacggaAaacugauaaggg...	2	1	Fm2
....uggacggagaacugauaNggg...	1	1	Fm2
....uggacggGgaacugauaaggg...	4	1	Fm2
....uggacggagaacAgauaaggg...	3	1	Fm2
....uggacggagaacugauaGggg...	193	1	Fm2
....uggacCgagaacugauaaggg...	1	1	Fm2
....uggacggagaacugCuaaggg...	2	1	Fm2
....uggacggagaacuCauaaggg...	2	1	Fm2
....uggacggagGacugauaaggg...	27	1	Fm2
....uggacggagaacugauaaggC...	16	1	Fm2
....uggacggagaacugauaaAgg...	3	1	Fm2
....Nggacggagaacugauaaggg...	16	1	Fm2
....ugAacggagaacugauaaggg...	8	1	Fm2
....uggacggagaacugauUaggg...	56	1	Fm2
....uAgacggagaacugauaaggg...	20	1	Fm2
....uggacggagaaAugauaaggg...	1	1	Fm2
....uggacggagaacugauaaUgg...	13	1	Fm2
....uggCcggagaacugauaaggg...	4	1	Fm2
....uggacggagaacuAauaaggg...	3	1	Fm2
....uggacggagaacugauaagCg...	2	1	Fm2
....uggaGggagaacugauaaggg...	5	1	Fm2
....uggacgUagaacugauaaggg...	15	1	Fm2
....uggacggagaaGugauaaggg...	1	1	Fm2
....Aggacggagaacugauaaggg...	22	1	Fm2
....uggacggagaCcugauaaggg...	67	1	Fm2
....uggacggagaacugauGaggg...	52	1	Fm2
....uggacggagaacugauaaggU...	824	1	Fm2
....uggacUgagaacugauaaggg...	9	1	Fm2
....uggacggagaacCgauaaggg...	7	1	Fm2
....uggacggagaacugauaagAg...	8	1	Fm2
....uggUcggagaacugauaaggg...	4	1	Fm2
....uggacgCagaacugauaaggg...	3	1	Fm2

Star **Mature**

gaauuuguccgaaagcggguccucgucauuuuuccgggccuguuuuuauacccuacuggacggagaacugauaagggcucgaauaggaccaagcaacauuugacguuaucugu			
....uggacggagUacugauaaggg....................................	16	1	Fm2
....uggacggagaacuUauaaggg....................................	2	1	Fm2
....uggacggagaacugauaaCgg....................................	3	1	Fm2
....uggacggagaacugauaUggg....................................	112	1	Fm2
....uggacggagaacugauaaggA....................................	6724	1	Fm2
....uggGcggagaacugauaaggg....................................	5	1	Fm2
....uggacggagaacugaAaaggg....................................	2	1	Fm2
....ugUacggagaacugauaaggg....................................	5	1	Fm2
....uggacggagaGcugauaaggg....................................	127	1	Fm2
....uggacggagaaUugauaaggg....................................	15	1	Fm2
....uggacggagaacugauaagUg....................................	9	1	Fm2
....uggacggaUaacugauaaggg....................................	5	1	Fm2
....uggacggagaacugGuaaggg....................................	4	1	Fm2
....Gggacggagaacugauaaggg....................................	4	1	Fm2
....uggaAggagaacugauaaggg....................................	2	1	Fm2
....uggacggagCacugauaaggg....................................	8	1	Fm2
....uggacggagaacugaGaaggg....................................	1	1	Fm2
....uggacggagaacCgauaagggc...................................	29	1	Fm2
....Cggacggagaacugauaagggc...................................	37	1	Fm2
....Aggacggagaacugauaagggc...................................	62	1	Fm2
....uggacggagaacugauaaCggc...................................	8	1	Fm2
....uggacggagaaGugauaagggc...................................	6	1	Fm2
....ugAacggagaacugauaagggc...................................	70	1	Fm2
....uggacggagaacuCauaagggc...................................	16	1	Fm2
....uggacggagaacugaGaagggc...................................	9	1	Fm2
....ugNacggagaacugauaagggc...................................	2	1	Fm2
....uggacggagaacugauaUgggc...................................	904	1	Fm2
....uggacggagaacugauaaggCc...................................	31	1	Fm2
....uggacggCgaacugauaagggc...................................	50	1	Fm2
....uggacggagaacugauaaAggc...................................	40	1	Fm2
....uggaUggagaacugauaagggc...................................	84	1	Fm2
....uggacggagaCcugauaagggc...................................	221	1	Fm2
....uggacggagaacugauCagggc...................................	583	1	Fm2
....uggacAgagaacugauaagggc...................................	38	1	Fm2
....ugUacggagaacugauaagggc...................................	54	1	Fm2
....uggacgCagaacugauaagggc...................................	27	1	Fm2
....uggacggGgaacugauaagggc...................................	83	1	Fm2
....uggacCgagaacugauaagggc...................................	10	1	Fm2
....uggacggagaacugauaagggU..................................	2043	1	Fm2
....uggacggagaacugauaCgggc...................................	1038	1	Fm2
....uggacggagaacAgauaagggc...................................	7	1	Fm2
....uggacggaAaacugauaagggc...................................	15	1	Fm2
....uUgacggagaacugauaagggc...................................	77	1	Fm2
....uCgacggagaacugauaagggc...................................	38	1	Fm2
....uAgacggagaacugauaagggc...................................	133	1	Fm2
....uggacggagaacugauaagggG..................................	452	1	Fm2
....uggCcggagaacugauaagggc...................................	44	1	Fm2
....uggacggagaacugauaaggUc...................................	78	1	Fm2
....uggacggagaacugauaagggA..................................	13052	1	Fm2
....uggacgUagaacugauaagggc...................................	60	1	Fm2
....ugCacggagaacugauaagggc...................................	28	1	Fm2
....uggacggagaacugCuaagggc...................................	7	1	Fm2
....uggaGggagaacugauaagggc...................................	13	1	Fm2
....uggacggagaacugauUagggc...................................	345	1	Fm2
....uggacggagaacugauGagggc...................................	490	1	Fm2
....uggacggagGacugauaagggc...................................	1032	1	Fm2
....uggacggaCaacugauaagggc...................................	11	1	Fm2
....uggacggagaacugauaagUgc...................................	78	1	Fm2
....uggUcggagaacugauaagggc...................................	43	1	Fm2
....uggacggagaacugauaaggAc...................................	83	1	Fm2
....Nggacggagaacugauaagggc...................................	143	1	Fm2
....uggaAggagaacugauaagggc...................................	32	1	Fm2
....uggacggagaacugauaagCgc...................................	17	1	Fm2
....uggacUgagaacugauaagggc...................................	58	1	Fm2
....uggacggagaacugauaagggc...................................	272144	0	Fm2
....uggGcggagaacugauaagggc...................................	55	1	Fm2
....uggacggagaacuUauaagggc...................................	30	1	Fm2
....uNgacggagaacugauaagggc...................................	3	1	Fm2
....uggacggagaacugauaGgggc...................................	6080	1	Fm2
....uggacggagUacugauaagggc...................................	107	1	Fm2

```
                    Star                         Mature
gaauuuguccgaaagcggguccucgucauuuuuccgggccuguuuuuauacccuacuggacggagaacugauaagggcucgaauaggaccaagcaacauuugacguuaucugu
........................................................uggacggagaacuAauaagggc...................................    64      1      Fm2
........................................................uggacggagaacugauaaUggc...................................    66      1      Fm2
........................................................uggacggagaaAugauaagggc...................................    5       1      Fm2
........................................................uggacggagCacugauaagggc...................................    56      1      Fm2
........................................................uggacggagaaUugauaagggc...................................    70      1      Fm2
........................................................uggacggagaacugGuaagggc...................................    26      1      Fm2
........................................................uggacggUgaacugauaagggc...................................    161     1      Fm2
........................................................uggacggagaacugauaagAgc...................................    38      1      Fm2
........................................................uggacggagaacugUuaagggc...................................    40      1      Fm2
........................................................uggacggagaacugaAaagggc...................................    7       1      Fm2
........................................................uggacgNagaacugauaagggc...................................    16      1      Fm2
........................................................Gggacggagaacugauaagggc...................................    7       1      Fm2
........................................................uggacggagaacugaCaagggc...................................    22      1      Fm2
........................................................uggacggagaacGgauaagggc...................................    7       1      Fm2
........................................................uggacggagaGcugauaagggc...................................    478     1      Fm2
........................................................uggacNgagaacugauaagggc...................................    34      1      Fm2
........................................................uggacgAagaacugauaagggc...................................    32      1      Fm2
........................................................uggacggaUaacugauaagggc...................................    55      1      Fm2
........................................................ugUacggagaacugauaagggcu..................................    2       1      Fm2
........................................................uggacggGgaacugauaagggcu..................................    1       1      Fm2
........................................................uggaAggagaacugauaagggcu..................................    1       1      Fm2
........................................................uggacggagaacugauaagggcG..................................    874     1      Fm2
........................................................uNgacggagaacugauaagggcu..................................    1       1      Fm2
........................................................uggacggagaacugauaagggcC..................................    179     1      Fm2
........................................................uggacggagaacugauaagggUu..................................    448     1      Fm2
........................................................uCgacggagaacugauaagggcu..................................    1       1      Fm2
........................................................uggacggagaacuAauaagggcu..................................    2       1      Fm2
........................................................uggacggagGacugauaagggcu..................................    37      1      Fm2
........................................................uggacggagaacugauCagggcu..................................    29      1      Fm2
........................................................uggacggagaacugauaagCgcu..................................    1       1      Fm2
........................................................uggacggagaCcugauaagggcu..................................    14      1      Fm2
........................................................uggacggagaacAgauaagggcu..................................    1       1      Fm2
........................................................uggacggagaacugauaagggAu..................................    634     1      Fm2
........................................................Aggacggagaacugauaagggcu..................................    2       1      Fm2
........................................................uggacggagaacugauaagUgcu..................................    4       1      Fm2
........................................................uggacggagaacuCauaagggcu..................................    1       1      Fm2
........................................................uggacggagaacugauaaggUcu..................................    5       1      Fm2
........................................................uggacggagCacugauaagggcu..................................    3       1      Fm2
........................................................uggaUggagaacugauaagggcu..................................    2       1      Fm2
........................................................Cggacggagaacugauaagggcu..................................    4       1      Fm2
........................................................uggacAgagaacugauaagggcu..................................    3       1      Fm2
........................................................uggacNgagaacugauaagggcu..................................    2       1      Fm2
........................................................uggacUgagaacugauaagggcu..................................    4       1      Fm2
........................................................uggacgAagaacugauaagggcu..................................    3       1      Fm2
........................................................uggacggaUaacugauaagggcu..................................    2       1      Fm2
........................................................uggacgNagaacugauaagggcu..................................    1       1      Fm2
........................................................uggacggagaaUugauaagggcu..................................    2       1      Fm2
........................................................uggacggagaacugaAaagggcu..................................    2       1      Fm2
........................................................uggacggagaacugauaGgggcu..................................    292     1      Fm2
........................................................uggacggagaacugauaCgggcu..................................    44      1      Fm2
........................................................uggGcggagaacugauaagggcu..................................    1       1      Fm2
........................................................uggacggagaacugauaaUggcu..................................    4       1      Fm2
........................................................uggacggagaacugauaagAgcu..................................    2       1      Fm2
........................................................uUgacggagaacugauaagggcu..................................    8       1      Fm2
........................................................uggacggagaacugauaUgggcu..................................    46      1      Fm2
........................................................uggacggagaacugauaaAggcu..................................    11      1      Fm2
........................................................uggacggagaacuUauaagggcu..................................    2       1      Fm2
........................................................uggacggagaacugauaaggAcu..................................    4       1      Fm2
........................................................uggacggagaacugUuaagggcu..................................    1       1      Fm2
........................................................uggacggagaGcugauaagggcu..................................    18      1      Fm2
........................................................uggacggCgaacugauaagggcu..................................    2       1      Fm2
........................................................uggacgUagaacugauaagggcu..................................    3       1      Fm2
........................................................uggacggUgaacugauaagggcu..................................    8       1      Fm2
........................................................uggacggagaacugauUagggcu..................................    20      1      Fm2
........................................................uggCcggagaacugauaagggcu..................................    1       1      Fm2
........................................................uggaGggagaacugauaagggcu..................................    1       1      Fm2
........................................................uggacggagaacugauaaggCcu..................................    3       1      Fm2
........................................................ugCacggagaacugauaagggcu..................................    1       1      Fm2
........................................................uggacggagaacugauGagggcu..................................    32      1      Fm2
........................................................uggacggagaacugauaagggcu..................................    12261   0      Fm2
```

```
                    Star                           Mature
gaauuuguccgaaagcggguccucgucauuuuuccgggccuguuuuuauacccuacuggacggagaacugauaagggcucgaauaggaccaagcaacauuugacguuaucugu
........................................................uggacggagUacugauaagggcu..................................  3    1  Fm2
........................................................uggacggagaacugGuaagggcu..................................  2    1  Fm2
........................................................Nggacggagaacugauaagggcu..................................  4    1  Fm2
........................................................uggUcggagaacugauaagggcu..................................  3    1  Fm2
........................................................uAgacggagaacugauaagggcu..................................  5    1  Fm2
........................................................uggacggagaacugauaGgggcuc.................................  2    1  Fm2
........................................................uggacggagaacugauaCgggcuc.................................  1    1  Fm2
........................................................uggacggagaacugauaagggcuc.................................  108  0  Fm2
........................................................uggacggagaacugauaagggcuG.................................  31   1  Fm2
........................................................uggacggagaacugauaagggcuA.................................  113  1  Fm2
.........................................................ggacggagaacugauaaA......................................  2    1  Fm2
.........................................................ggacggagaacugauaaU......................................  5    1  Fm2
.........................................................ggacggagaacugauaag......................................  3    0  Fm2
.........................................................ggacggagaacugauaagg.....................................  30   0  Fm2
.........................................................ggacggagGacugauaagg.....................................  1    1  Fm2
.........................................................ggacggagaacugauaagU.....................................  1    1  Fm2
.........................................................ggacggagaacGgauaagg.....................................  1    1  Fm2
.........................................................Ugacggagaacugauaagg.....................................  2    1  Fm2
.........................................................ggacggagaacugauaagA.....................................  2    1  Fm2
.........................................................ggacggagaacugauaGgg.....................................  1    1  Fm2
.........................................................ggacggagaacugauaaggg....................................  39   0  Fm2
.........................................................ggacggagaacugauaagUg....................................  1    1  Fm2
.........................................................ggacggagaacugauaagAg....................................  1    1  Fm2
.........................................................Ugacggagaacugauaaggg....................................  5    1  Fm2
.........................................................ggacggagaacugauaaggA....................................  8    1  Fm2
.........................................................ggacggagaacugauaaggAc...................................  3    1  Fm2
.........................................................ggacggagaacugauaagggU...................................  1    1  Fm2
.........................................................ggacggagaacugauaGgggc...................................  6    1  Fm2
.........................................................ggacggagaaAugauaagggc...................................  1    1  Fm2
.........................................................ggacggNgaacugauaagggc...................................  1    1  Fm2
.........................................................ggacggagUacugauaagggc...................................  1    1  Fm2
.........................................................ggacggagaacuUauaagggc...................................  1    1  Fm2
.........................................................ggacggagCacugauaagggc...................................  3    1  Fm2
.........................................................ggacggagaacugauaagggc...................................  191  0  Fm2
.........................................................ggacggagaacugauaaggCc...................................  1    1  Fm2
.........................................................ggacggaAaacugauaagggc...................................  1    1  Fm2
.........................................................ggacggagaaUugauaagggc...................................  2    1  Fm2
.........................................................ggacggagaacuAauaagggc...................................  2    1  Fm2
.........................................................ggacggagaacugauaagCgc...................................  1    1  Fm2
.........................................................Ugacggagaacugauaagggc...................................  13   1  Fm2
.........................................................Agacggagaacugauaagggc...................................  1    1  Fm2
.........................................................ggacgUagaacugauaagggc...................................  1    1  Fm2
.........................................................ggacggUgaacugauaagggc...................................  1    1  Fm2
.........................................................ggacggagaacugauaagggA...................................  22   1  Fm2
.........................................................ggacggCgaacugauaagggcu..................................  1    1  Fm2
.........................................................ggacggagaacugauaagggcu..................................  8    0  Fm2
.........................................................ggacggagaacugauaagggcG..................................  1    1  Fm2
.........................................................ggacggagaacugauaagggcuc.................................  2    0  Fm2
.........................................................ggacggagaacuUauaagggcuc.................................  1    1  Fm2
..........................................................gacggagaacugauaagg.....................................  1    0  Fm2
............................................................Uggagaacugauaagggc...................................  1    1  Fm2
............................................................cggagaacugauaagggc...................................  5    0  Fm2

...................uccucgucauuuuuccgggccu........................................................................  1    0  Mm3
....................ccucgucauuuuuccgggccu........................................................................  1    0  Mm3
....................ccucgucauuuuuccgggccugG......................................................................  2    1  Mm3
....................ccuUgucauuuuuccgggccugu......................................................................  1    1  Mm3
....................ccucgucauuuuuccgggccugu......................................................................  31   0  Mm3
....................ccucgucauuuuuccggUccugu......................................................................  2    1  Mm3
.....................cucgucauuuuuccgggccugu......................................................................  2    0  Mm3
..........................................uuuuuauacccuacuggacggagaac.............................................  1    0  Mm3
...........................................uuuuauacccuacuggacggagaac.............................................  1    0  Mm3
...........................................uuuuauacccuacuggacggagaacugau.........................................  2    0  Mm3
......................................................Ucuggacggagaacugauaagggc...................................  6    1  Mm3
.......................................................cuggacggagaacugaua........................................  1    0  Mm3
.......................................................cuggacggagaacugauaa.......................................  3    0  Mm3
.......................................................cuggacggagaacugauaag......................................  13   0  Mm3
.......................................................cuggacggagaacugauaaA......................................  5    1  Mm3
.......................................................cuggacggagaacugauUagg.....................................  1    1  Mm3
.......................................................cuggacggagaacugauaagg.....................................  18   0  Mm3
```

```
                    Star                                Mature
gaauuuguccgaaagcggguccucgucauuuuuccgggccuguuuuuauacccuacuggacggagaacugauaagggcucgaauaggaccaagcaacauuugacguuaucugu
.......................................................cuggaNggagaacugauaagg.....................................   1     1   Mm3
.......................................................cuggacggagaacugauaagA.....................................   9     1   Mm3
.......................................................cuggacggagaacugauaaggA....................................   4     1   Mm3
.......................................................cuggacggagaacugauaaggU....................................   3     1   Mm3
.......................................................cuggacggagaacugauCaggg....................................   2     1   Mm3
.......................................................cuggacggagaacugauaaggC....................................   1     1   Mm3
.......................................................cuggacggagaacugauaGggg....................................   1     1   Mm3
.......................................................cuggacggagaacugauaaggg....................................   33    0   Mm3
.......................................................cuggacggagaacugauaagggA...................................   2     1   Mm3
.......................................................cAggacggagaacugauaagggc...................................   2     1   Mm3
.......................................................cuggacggagaacugauaagggc...................................   112   0   Mm3
.......................................................cuggacggagaacugauaagggU...................................   2     1   Mm3
.......................................................cuggCcggagaacugauaagggc...................................   1     1   Mm3
.......................................................cuggacggagaacugauaagggcu..................................   10    0   Mm3
........................................................uggacggagaacugauaG.......................................   13    1   Mm3
........................................................uggacggagaacugaCaa.......................................   2     1   Mm3
........................................................uggacggagaCcugauaa.......................................   1     1   Mm3
........................................................uggacggagaaUugauaa.......................................   1     1   Mm3
........................................................uggacggagaacugauUa.......................................   5     1   Mm3
........................................................uggacggagaacugauCa.......................................   6     1   Mm3
........................................................uggacggUgaacugauaa.......................................   1     1   Mm3
........................................................uggaUggagaacugauaa.......................................   2     1   Mm3
........................................................uggacggagCacugauaa.......................................   1     1   Mm3
........................................................uggacggagaGcugauaa.......................................   3     1   Mm3
........................................................uggacUgagaacugauaa.......................................   1     1   Mm3
........................................................uggacggagaacCgauaa.......................................   1     1   Mm3
........................................................uggacggaCaacugauaa.......................................   1     1   Mm3
........................................................uggacggagaacugaAaa.......................................   66    1   Mm3
........................................................uggacggagaacugauaC.......................................   4     1   Mm3
........................................................uggCcggagaacugauaa.......................................   1     1   Mm3
........................................................uggacggagaacugauGa.......................................   2     1   Mm3
........................................................uggacggagaacugauaU.......................................   62    1   Mm3
........................................................Nggacggagaacugauaa.......................................   2     1   Mm3
........................................................Aggacggagaacugauaa.......................................   2     1   Mm3
........................................................uggacggagaacugauaa.......................................   2074  0   Mm3
........................................................uggacggaUaacugauaag......................................   2     1   Mm3
........................................................uggacggagaCcugauaag......................................   14    1   Mm3
........................................................uggacggagaacugUuaag......................................   1     1   Mm3
........................................................uggGcggagaacugauaag......................................   1     1   Mm3
........................................................uggacggagaacugauaCg......................................   30    1   Mm3
........................................................ugAacggagaacugauaag......................................   4     1   Mm3
........................................................uggacggagaacugauUag......................................   14    1   Mm3
........................................................Cggacggagaacugauaag......................................   2     1   Mm3
........................................................uUgacggagaacugauaag......................................   2     1   Mm3
........................................................uggacUgagaacugauaag......................................   2     1   Mm3
........................................................uggacggGgaacugauaag......................................   3     1   Mm3
........................................................uggacggagaacugaAaag......................................   3     1   Mm3
........................................................uggacggagaacuUauaag......................................   4     1   Mm3
........................................................Nggacggagaacugauaag......................................   3     1   Mm3
........................................................uggacggagaacugauaaU......................................   1351  1   Mm3
........................................................uggacAgagaacugauaag......................................   4     1   Mm3
........................................................uggacggagCacugauaag......................................   1     1   Mm3
........................................................Aggacggagaacugauaag......................................   11    1   Mm3
........................................................uggacggagGacugauaag......................................   9     1   Mm3
........................................................uggacggagaacugauaUg......................................   30    1   Mm3
........................................................uggacggagaacuAauaag......................................   4     1   Mm3
........................................................uAgacggagaacugauaag......................................   3     1   Mm3
........................................................uggacggagaacugCuaag......................................   1     1   Mm3
........................................................uggacggagaaAugauaag......................................   1     1   Mm3
........................................................uggUcggagaacugauaag......................................   1     1   Mm3
........................................................uggacggagaacugaCaag......................................   1     1   Mm3
........................................................uggacggagaacCgauaag......................................   1     1   Mm3
........................................................uggacggagaacugauGag......................................   10    1   Mm3
........................................................uggacggagUacugauaag......................................   4     1   Mm3
........................................................uggacggagaaUugauaag......................................   2     1   Mm3
........................................................uggacggagaacugauaaC......................................   15    1   Mm3
........................................................uggacggagaGcugauaag......................................   30    1   Mm3
........................................................uggacggagaacugauCag......................................   11    1   Mm3
........................................................uggacggagaacugauaaA......................................   5374  1   Mm3
........................................................uggacggagaacugauaag......................................   8431  0   Mm3
```

Star Mature

```
gaauuuguccgaaagcggguccucgucauuuuuccgggccuguuuuuauacccuacuggacggagaacugauaagggcucgaauaggaccaagcaacauuugacguuaucugu
........................................................uggacggUgaacugauaag......................................    3      1    Mm3
........................................................uggaAggagaacugauaag......................................    1      1    Mm3
........................................................uggacggagaacugauaGg.....................................    40     1    Mm3
........................................................uggCcggagaacugauaag......................................    1      1    Mm3
........................................................uggacggagaacugauUagg.....................................    12     1    Mm3
........................................................uggacggagaaUugauaagg.....................................    3      1    Mm3
........................................................uggaUggagaacugauaagg.....................................    7      1    Mm3
........................................................uggacggagUacugauaagg.....................................    2      1    Mm3
........................................................uggacggagaaAugauaagg.....................................    1      1    Mm3
........................................................uggacggagaacugauaagg.....................................    14584  0    Mm3
........................................................uggacUgagaacugauaagg.....................................    11     1    Mm3
........................................................uggacggagaacugauaGgg.....................................    91     1    Mm3
........................................................uggacggagaacuUauaagg.....................................    1      1    Mm3
........................................................uggacgAagaacugauaagg.....................................    2      1    Mm3
........................................................Cggacggagaacugauaagg.....................................    4      1    Mm3
........................................................uggacggGgaacugauaagg.....................................    2      1    Mm3
........................................................uggacCgagaacugauaagg.....................................    1      1    Mm3
........................................................Nggacggagaacugauaagg.....................................    6      1    Mm3
........................................................uggacggagaacugauaaUg.....................................    2      1    Mm3
........................................................uggacggagaacugauaagA.....................................    3650   1    Mm3
........................................................uggacgUagaacugauaagg.....................................    1      1    Mm3
........................................................uggacggagaGcugauaagg.....................................    140    1    Mm3
........................................................uggCcggagaacugauaagg.....................................    3      1    Mm3
........................................................ugUacggagaacugauaagg.....................................    3      1    Mm3
........................................................uggacggagGacugauaagg.....................................    27     1    Mm3
........................................................ugCacggagaacugauaagg.....................................    2      1    Mm3
........................................................uggacggCgaacugauaagg.....................................    2      1    Mm3
........................................................uggacggagaacuAauaagg.....................................    1      1    Mm3
........................................................uNgacggagaacugauaagg.....................................    1      1    Mm3
........................................................uggacgCagaacugauaagg.....................................    3      1    Mm3
........................................................uggacggagaacugauCagg.....................................    31     1    Mm3
........................................................Gggacggagaacugauaagg.....................................    1      1    Mm3
........................................................uggacggagaacugauaCgg.....................................    58     1    Mm3
........................................................uAgacggagaacugauaagg.....................................    11     1    Mm3
........................................................uggacggaAaacugauaagg.....................................    2      1    Mm3
........................................................uggacggagaCcugauaagg.....................................    76     1    Mm3
........................................................uggacggagaacugauaagC.....................................    10     1    Mm3
........................................................uggacggagCacugauaagg.....................................    5      1    Mm3
........................................................uUgacggagaacugauaagg.....................................    7      1    Mm3
........................................................uggacAgagaacugauaagg.....................................    2      1    Mm3
........................................................uggGcggagaacugauaagg.....................................    5      1    Mm3
........................................................uggacggagaacGgauaagg.....................................    2      1    Mm3
........................................................uggacggUgaacugauaagg.....................................    6      1    Mm3
........................................................uggacggaUaacugauaagg.....................................    1      1    Mm3
........................................................Aggacggagaacugauaagg.....................................    140    1    Mm3
........................................................uggUcggagaacugauaagg.....................................    4      1    Mm3
........................................................uggacggagaacugUuaagg.....................................    3      1    Mm3
........................................................uggacggagaacCgauaagg.....................................    1      1    Mm3
........................................................uggacggaCaacugauaagg.....................................    1      1    Mm3
........................................................uggacggagaacugauaaAg.....................................    91     1    Mm3
........................................................uggacNgagaacugauaagg.....................................    3      1    Mm3
........................................................uggacggagaacugGuaagg.....................................    1      1    Mm3
........................................................uggacggagaacugauaagU.....................................    617    1    Mm3
........................................................uggacggagaacugauaUgg.....................................    64     1    Mm3
........................................................uggacggagaacugauGagg.....................................    21     1    Mm3
........................................................uCgacggagaacugauaagg.....................................    1      1    Mm3
........................................................uggacggUgaacugauaaggg....................................    15     1    Mm3
........................................................ugUacggagaacugauaaggg....................................    3      1    Mm3
........................................................uggacAgagaacugauaaggg....................................    5      1    Mm3
........................................................uggacggagaacugaCaaggg....................................    1      1    Mm3
........................................................uggacggagaacugauaaggC....................................    89     1    Mm3
........................................................uggacggagaacugaAaaggg....................................    1      1    Mm3
........................................................uggacggagaacugUuaaggg....................................    5      1    Mm3
........................................................uggacggagaacugauaaggU....................................    657    1    Mm3
........................................................Nggacggagaacugauaaggg....................................    13     1    Mm3
........................................................uggacggaAaacugauaaggg....................................    1      1    Mm3
........................................................uggacggagaacugauaGggg....................................    205    1    Mm3
........................................................uggacggagaacuCauaaggg....................................    2      1    Mm3
........................................................Aggacggagaacugauaaggg....................................    102    1    Mm3
........................................................uggacggagaacuUauaaggg....................................    5      1    Mm3
```

```
                    Star                                  Mature
gaauuuguccgaaagcggguccucgucauuuuuccgggccuguuuuuauacccuacuggacggagaacugauaagggcucgaauaggaccaagcaacauuugacguuaucugu
........................................................uggacggagaacugauaagCg....................................    1      1  Mm3
........................................................uAgacggagaacugauaaggg....................................    9      1  Mm3
........................................................uggacggagaacuAauaaggg....................................    6      1  Mm3
........................................................uggacCgagaacugauaaggg....................................    2      1  Mm3
........................................................uggacggaUaacugauaaggg....................................    6      1  Mm3
........................................................uggacggagaacugauCaggg....................................    60     1  Mm3
........................................................uggacggagaacugauaagUg....................................    2      1  Mm3
........................................................uggacggagaacugauaaggg....................................    27231  0  Mm3
........................................................uggacggagaGcugauaaggg....................................    145    1  Mm3
........................................................uUgacggagaacugauaaggg....................................    5      1  Mm3
........................................................uggacggagCacugauaaggg....................................    5      1  Mm3
........................................................uggacggagaacAgauaaggg....................................    3      1  Mm3
........................................................uggacggCgaacugauaaggg....................................    4      1  Mm3
........................................................uggacggagGacugauaaggg....................................    35     1  Mm3
........................................................uggCcggagaacugauaaggg....................................    3      1  Mm3
........................................................Gggacggagaacugauaaggg....................................    1      1  Mm3
........................................................uggacggagaaAugauaaggg....................................    2      1  Mm3
........................................................uggGcggagaacugauaaggg....................................    6      1  Mm3
........................................................uggaAggagaacugauaaggg....................................    2      1  Mm3
........................................................uggacggagaacugauaCggg....................................    118    1  Mm3
........................................................uggacgNagaacugauaaggg....................................    2      1  Mm3
........................................................uggacggaCaacugauaaggg....................................    1      1  Mm3
........................................................uggacNgagaacugauaaggg....................................    2      1  Mm3
........................................................uggacggagaacugauUaggg....................................    38     1  Mm3
........................................................uggacggagaacugauGaggg....................................    40     1  Mm3
........................................................uggacggagaacugauaaUgg....................................    3      1  Mm3
........................................................uggacUgagaacugauaaggg....................................    13     1  Mm3
........................................................uggacggagaacugGuaaggg....................................    1      1  Mm3
........................................................uggacggagaacugauaUggg....................................    74     1  Mm3
........................................................Cggacggagaacugauaaggg....................................    11     1  Mm3
........................................................uggacggagaaUugauaaggg....................................    7      1  Mm3
........................................................uggacggagaacugauaaggA....................................    4536   1  Mm3
........................................................uggaUggagaacugauaaggg....................................    3      1  Mm3
........................................................uggacggagaacCgauaaggg....................................    4      1  Mm3
........................................................uggacggagaacugauaagAg....................................    28     1  Mm3
........................................................uggacggGgaacugauaaggg....................................    11     1  Mm3
........................................................uggacggagUacugauaaggg....................................    9      1  Mm3
........................................................uggacggagaacugCuaaggg....................................    1      1  Mm3
........................................................uggacgAagaacugauaaggg....................................    5      1  Mm3
........................................................uggacggagaacugauaaAgg....................................    3      1  Mm3
........................................................uggacggagaCcugauaaggg....................................    71     1  Mm3
........................................................uggacgUagaacugauaaggg....................................    5      1  Mm3
........................................................ugAacggagaacugauaaggg....................................    7      1  Mm3
........................................................uCgacggagaacugauaaggg....................................    1      1  Mm3
........................................................uggUcggagaacugauaaggg....................................    3      1  Mm3
........................................................uggacggagaacugauaNgggc...................................    2      1  Mm3
........................................................ugCacggagaacugauaagggc...................................    24     1  Mm3
........................................................uggacggGgaacugauaagggc...................................    76     1  Mm3
........................................................Cggacggagaacugauaagggc...................................    55     1  Mm3
........................................................uggacggagaacugauaagUgc...................................    69     1  Mm3
........................................................uggacggagaCcugauaagggc...................................    247    1  Mm3
........................................................uggacggagCacugauaagggc...................................    54     1  Mm3
........................................................uggacggagaacuUauaagggc...................................    28     1  Mm3
........................................................Aggacggagaacugauaagggc...................................    284    1  Mm3
........................................................uggacggagaacugauaagggG...................................    144    1  Mm3
........................................................uggacggagaacugauaCgggc...................................    1119   1  Mm3
........................................................uggacggagaacugaAaagggc...................................    10     1  Mm3
........................................................uggacggagaacugauaaggAc...................................    60     1  Mm3
........................................................uggacggagaacugauaagggU...................................    2911   1  Mm3
........................................................uggacggagaacugaGaagggc...................................    2      1  Mm3
........................................................uggacggagaacugauGagggc...................................    556    1  Mm3
........................................................uggGcggagaacugauaagggc...................................    58     1  Mm3
........................................................uggUcggagaacugauaagggc...................................    42     1  Mm3
........................................................uggacggagaacCgauaagggc...................................    35     1  Mm3
........................................................uggacUgagaacugauaagggc...................................    32     1  Mm3
........................................................uggacggagaacugauaagAgc...................................    49     1  Mm3
........................................................uggacggagaacugauaaUggc...................................    50     1  Mm3
........................................................uggacggagaaGugauaagggc...................................    6      1  Mm3
........................................................uggCcggagaacugauaagggc...................................    70     1  Mm3
........................................................uggaGggagaacugauaagggc...................................    14     1  Mm3
```

Star Mature

gaauuuguccgaaagcgggu**ccucgucauuuuuccgggccugu**uuuuauacccuac**uggacggagaacugauaagggc**ucgaauaggaccaagcaacauuugacguuaucugu			
....uggacggagaacGgauaagggc...................................	12	1	Mm3
....uggacggagaacugauaagggc...................................	293785	0	Mm3
....uggacgAagaacugauaagggc...................................	36	1	Mm3
....uggacgUagaacugauaagggc...................................	53	1	Mm3
....uggacggagaacugauaaAggc...................................	31	1	Mm3
....uggacggagaacugauaGgggc...................................	2855	1	Mm3
....uggacggagaacugGuaagggc...................................	29	1	Mm3
....uggacgCagaacugauaagggc...................................	17	1	Mm3
....uggacAgagaacugauaagggc...................................	50	1	Mm3
....uggacggagaacuCauaagggc...................................	19	1	Mm3
....uggacggagaaAugauaagggc...................................	9	1	Mm3
....uggacggagaacugauaagCgc...................................	22	1	Mm3
....uggaAggagaacugauaagggc...................................	29	1	Mm3
....uggacggagaacugauCagggc...................................	629	1	Mm3
....uggaUggagaacugauaagggc...................................	74	1	Mm3
....Gggacggagaacugauaagggc...................................	15	1	Mm3
....uggacCgagaacugauaagggc...................................	7	1	Mm3
....uggacggagaacugCuaagggc...................................	9	1	Mm3
....uggacggagaacugauaagggA...................................	8184	1	Mm3
....uggacggagaacugauaUgggc...................................	986	1	Mm3
....uggacggaUaacugauaagggc...................................	47	1	Mm3
....uggacggagaacugauUagggc...................................	428	1	Mm3
....uggacggUgaacugauaagggc...................................	168	1	Mm3
....uggacggagaaUugauaagggc...................................	74	1	Mm3
....uggacggagUacugauaagggc...................................	126	1	Mm3
....ugAacggagaacugauaagggc...................................	81	1	Mm3
....uggacggagaacugauaaCggc...................................	7	1	Mm3
....ugNacggagaacugauaagggc...................................	2	1	Mm3
....uggacggagaGcugauaagggc...................................	541	1	Mm3
....uggacggaCaacugauaagggc...................................	13	1	Mm3
....Nggacggagaacugauaagggc...................................	148	1	Mm3
....uggacggaAaacugauaagggc...................................	19	1	Mm3
....uggacggagaacugauaaggUc...................................	74	1	Mm3
....uggacggagaacAgauaagggc...................................	17	1	Mm3
....uUgacggagaacugauaagggc...................................	58	1	Mm3
....uggacNgagaacugauaagggc...................................	38	1	Mm3
....uCgacggagaacugauaagggc...................................	32	1	Mm3
....uggacggagaacugUuaagggc...................................	56	1	Mm3
....uggacggagaacugauaaggCc...................................	161	1	Mm3
....uggacggagaacugaCaagggc...................................	20	1	Mm3
....uggacgNagaacugauaagggc...................................	22	1	Mm3
....ugUacggagaacugauaagggc...................................	39	1	Mm3
....uNgacggagaacugauaagggc...................................	3	1	Mm3
....uggacggagaacuAauaagggc...................................	60	1	Mm3
....uggacggCgaacugauaagggc...................................	43	1	Mm3
....uAgacggagaacugauaagggc...................................	111	1	Mm3
....uggacggagGacugauaagggc...................................	464	1	Mm3
....uggacggagaacuCauaagggcu..................................	1	1	Mm3
....uggacggagaacuUauaagggcu..................................	1	1	Mm3
....uggacggagaacugauGagggcu..................................	33	1	Mm3
....uggacggagUacugauaagggcu..................................	9	1	Mm3
....uggacggagaacugauaaAggcu..................................	1	1	Mm3
....uggacgNagaacugauaagggcu..................................	1	1	Mm3
....uggacggagaacugauaagggAu..................................	571	1	Mm3
....uggacggagaacugauUagggcu..................................	22	1	Mm3
....Cggacggagaacugauaagggcu..................................	1	1	Mm3
....uggUcggagaacugauaagggcu..................................	1	1	Mm3
....uggacggagaacAgauaagggcu..................................	2	1	Mm3
....uggacgCagaacugauaagggcu..................................	2	1	Mm3
....uggacggagaacugauaaggUcu..................................	2	1	Mm3
....uggacggagaacugauaaUggcu..................................	2	1	Mm3
....uNgacggagaacugauaagggcu..................................	1	1	Mm3
....uggacggagaacugaCaagggcu..................................	2	1	Mm3
....uggacggagaacugauaagggcG..................................	434	1	Mm3
....uggacggagaacuAauaagggcu..................................	1	1	Mm3
....uggacggagaacugauaUgggcu..................................	41	1	Mm3
....uggacggagaacugauaagUgcu..................................	3	1	Mm3
....uggacggagaacugauaaggAcu..................................	3	1	Mm3
....uggacggagCacugauaagggcu..................................	1	1	Mm3
....uggacggagaacCgauaagggcu..................................	1	1	Mm3

```
                    Star                              Mature
gaauuuguccgaaagcggguccucgucauuuuuccgggccuguuuuuauacccuacuggacggagaacugauaagggcucgaauaggaccaagcaacauuugacguuaucugu
........................................................uggacggagaacugauaagggGu..................................   3      1   Mm3
........................................................uggacgUagaacugauaagggcu..................................   1      1   Mm3
........................................................uggacggCgaacugauaagggcu..................................   3      1   Mm3
........................................................uggacggagaacugauaCgggcu..................................   63     1   Mm3
........................................................uggacggagaGcugauaagggcu..................................   20     1   Mm3
........................................................uggacggagaacugauaaggCcu..................................   3      1   Mm3
........................................................Nggacggagaacugauaagggcu..................................   5      1   Mm3
........................................................uggCcggagaacugauaagggcu..................................   2      1   Mm3
........................................................ugAacggagaacugauaagggcu..................................   3      1   Mm3
........................................................uggacggGgaacugauaagggcu..................................   8      1   Mm3
........................................................uggacggagaacugauaagggcC..................................   175    1   Mm3
........................................................uggacggagGacugauaagggcu..................................   22     1   Mm3
........................................................uggGcggagaacugauaagggcu..................................   1      1   Mm3
........................................................uAgacggagaacugauaagggcu..................................   7      1   Mm3
........................................................uggacggagaacugauaagggUu..................................   397    1   Mm3
........................................................ugCacggagaacugauaagggcu..................................   2      1   Mm3
........................................................uUgacggagaacugauaagggcu..................................   5      1   Mm3
........................................................uggacUgagaacugauaagggcu..................................   3      1   Mm3
........................................................uggacgAagaacugauaagggcu..................................   2      1   Mm3
........................................................uggacggagaacugauaGgggcu..................................   174    1   Mm3
........................................................uggacggUgaacugauaagggcu..................................   9      1   Mm3
........................................................ugUacggagaacugauaagggcu..................................   2      1   Mm3
........................................................uggacAgagaacugauaagggcu..................................   3      1   Mm3
........................................................uggacggagaacugUuaagggcu..................................   2      1   Mm3
........................................................uggacggagaacugGuaagggcu..................................   1      1   Mm3
........................................................uggacggagaacugauCagggcu..................................   42     1   Mm3
........................................................Aggacggagaacugauaagggcu..................................   16     1   Mm3
........................................................uggacggagaCcugauaagggcu..................................   18     1   Mm3
........................................................uggacggagaaUugauaagggcu..................................   4      1   Mm3
........................................................uggacggagaacugauaagggcu..................................   14479  0   Mm3
........................................................uggacNgagaacugauaagggcu..................................   3      1   Mm3
........................................................uggacggagaacugauaagggcCc.................................   1      1   Mm3
........................................................uggacggagaacugauaagggcuG.................................   10     1   Mm3
........................................................uggacggagaacugauaaggAcuc.................................   1      1   Mm3
........................................................uggacggagaacugauaagggcuA.................................   44     1   Mm3
........................................................uggacggagaacugauaagggcAc.................................   20     1   Mm3
........................................................uggacggagaacCgauaagggcuc.................................   1      1   Mm3
........................................................uggacggagaUcugauaagggcuc.................................   2      1   Mm3
........................................................uggacggagaacugauaagggcuc.................................   34     0   Mm3
.........................................................ggacggagaacugauaaA......................................   12     1   Mm3
.........................................................ggacggagaacugauaaU......................................   3      1   Mm3
.........................................................ggacggagaacugauaag......................................   18     0   Mm3
.........................................................ggacggagaacugauaagA.....................................   16     1   Mm3
.........................................................ggacggagaacugauaagg.....................................   33     0   Mm3
.........................................................ggacggagaCcugauaagg.....................................   1      1   Mm3
.........................................................ggacggCgaacugauaagg.....................................   1      1   Mm3
.........................................................ggacggagCacugauaagg.....................................   1      1   Mm3
.........................................................ggacggagaacugauaagC.....................................   1      1   Mm3
.........................................................Ugacggagaacugauaagg.....................................   1      1   Mm3
.........................................................Ugacggagaacugauaaggg....................................   2      1   Mm3
.........................................................ggacggagaacugauaaggg....................................   61     0   Mm3
.........................................................ggacggagaacugauaaggU....................................   1      1   Mm3
.........................................................ggacggagaacuUauaaggg....................................   1      1   Mm3
.........................................................ggacggagaacugauaaggA....................................   14     1   Mm3
.........................................................ggacggagCacugauaaggg....................................   2      1   Mm3
.........................................................ggacggagaGcugauaaggg....................................   2      1   Mm3
.........................................................ggacgCagaacugauaaggg....................................   1      1   Mm3
.........................................................ggaAggagaacugauaagggc...................................   1      1   Mm3
.........................................................ggacggagaacugauaaggUc...................................   1      1   Mm3
.........................................................ggacggaUaacugauaagggc...................................   1      1   Mm3
.........................................................ggacggagGacugauaagggc...................................   4      1   Mm3
.........................................................ggacggagaCcugauaagggc...................................   1      1   Mm3
.........................................................ggacggaAaacugauaagggc...................................   1      1   Mm3
.........................................................ggacggagaacugauaaAggc...................................   1      1   Mm3
.........................................................ggacggagaaGugauaagggc...................................   1      1   Mm3
.........................................................ggacggagaacugauaagggc...................................   621    0   Mm3
.........................................................ggacggagaaUugauaagggc...................................   1      1   Mm3
.........................................................Ngacggagaacugauaagggc...................................   1      1   Mm3
.........................................................ggacggagaacugauaagggA...................................   13     1   Mm3
.........................................................ggacggagaacugauaCgggc...................................   2      1   Mm3
```

Star   Mature

```
gaauuuguccgaaagcggguccucgucauuuuuccgggccuguuuuuauacccuacuggacggagaacugauaagggcucgaauaggaccaagcaacauuugacguuaucugu
.........................................................ggacggagaacugauCagggc...................................  4    1  Mm3
.........................................................ggacggUgaacugauaagggc...................................  1    1  Mm3
.........................................................ggacggagaacugauaUgggc...................................  1    1  Mm3
.........................................................ggacggagaacAgauaagggc...................................  1    1  Mm3
.........................................................ggacggagaacugaAaagggc...................................  1    1  Mm3
.........................................................ggacggagaacugauaagggU...................................  9    1  Mm3
.........................................................ggCcggagaacugauaagggc...................................  1    1  Mm3
.........................................................ggacggagaacugauaaggAc...................................  2    1  Mm3
.........................................................gUacggagaacugauaagggc...................................  2    1  Mm3
.........................................................ggacggagaaAugauaagggc...................................  1    1  Mm3
.........................................................ggacCgagaacugauaagggc...................................  1    1  Mm3
.........................................................ggacggagaacugauaaUggc...................................  1    1  Mm3
.........................................................ggacggagCacugauaagggc...................................  6    1  Mm3
.........................................................ggacggagaacugauaGgggc...................................  5    1  Mm3
.........................................................ggacggagaacugauaagggG...................................  1    1  Mm3
.........................................................Ugacggagaacugauaagggc...................................  12   1  Mm3
.........................................................ggacggagaacugauaagUgc...................................  1    1  Mm3
.........................................................ggacggagaacugauaagAgc...................................  2    1  Mm3
.........................................................ggacggagaacugauGagggc...................................  1    1  Mm3
.........................................................ggacggCgaacugauaagggcu..................................  1    1  Mm3
.........................................................ggacggagaacugauaagggcu..................................  38   0  Mm3
.........................................................ggacggagaacugauaagggUu..................................  1    1  Mm3
.........................................................ggacggagaacugauaaggUcu..................................  1    1  Mm3
.........................................................ggacggagaacugauCagggcu..................................  1    1  Mm3
..........................................................gacggagaacugauaagggc...................................  3    0  Mm3
...........................................................acggagaacugauaagggc...................................  2    0  Mm3

...................uccucgucauuuuuccgggccugu......................................................................  5    0  Mm2
...................uccAcgucauuuuuccgggccugu......................................................................  1    1  Mm2
...................uccucgucauuuuuccgggccuguu.....................................................................  1    0  Mm2
....................ccucgucauuuuuccgggccG........................................................................  1    1  Mm2
....................ccucgucauuuuuccgggccu........................................................................  2    0  Mm2
....................ccucgucauuuuuccgggccug.......................................................................  3    0  Mm2
....................ccucgucauuuuuccgggccugG......................................................................  7    1  Mm2
....................ccucgucauuuuuccgUgccugu......................................................................  3    1  Mm2
....................ccucgucauuuuuccggCccugu......................................................................  3    1  Mm2
....................ccucgucauuuuuccgggccugu......................................................................  338  0  Mm2
....................ccucgucauuuuuccgggccugC......................................................................  2    1  Mm2
....................ccucgucauuuuuccgCgccugu......................................................................  1    1  Mm2
....................ccucgucauuuuuccgAgccugu......................................................................  1    1  Mm2
....................ccucgucauuuuuccgggccuUu......................................................................  1    1  Mm2
....................ccucgucauuuuuccgggccAgu......................................................................  1    1  Mm2
....................ccucgucauuuuuccggUccugu......................................................................  1    1  Mm2
....................ccucgucauuuuuccggAccugu......................................................................  2    1  Mm2
....................ccucgucauuuuuccgggccuguu.....................................................................  37   0  Mm2
....................Ucucgucauuuuuccgggccuguu.....................................................................  1    1  Mm2
....................ccucgucauuuuuccgggccuguA.....................................................................  1    1  Mm2
....................ccucgucauuuuuccgggccuguuu....................................................................  10   0  Mm2
....................ccucgucauuuuuccgggccuguuuu...................................................................  1    0  Mm2
....................ccucgucauuuuuccgggccuguuuuuauacccuac.........................................................  1    0  Mm2
....................ccucgucauuuuuccgggccuguuuuuauacccuacuU.......................................................  1    1  Mm2
.....................cucgucauuuuuccgggccugu......................................................................  9    0  Mm2
.....................cucgucauuuuuccgggccuguuu....................................................................  1    0  Mm2
.............................uuuuccgggccuguuuuuauaccc............................................................  1    0  Mm2
...................................................ccuacuggacggagaacugauaagggc...................................  1    0  Mm2
......................................................acuggacggagaacugauaag......................................  1    0  Mm2
......................................................acuggacggagaacugauaagg.....................................  2    0  Mm2
......................................................Ucuggacggagaacugauaaggg....................................  2    1  Mm2
......................................................Ucuggacggagaacugauaagggc...................................  8    1  Mm2
......................................................Ucuggacggagaacugauaagggcu..................................  3    1  Mm2
.......................................................cuggacggagaacugaua........................................  1    0  Mm2
.......................................................cuggacggagaacugauaa.......................................  3    0  Mm2
.......................................................cuggacggagaacugauaaA......................................  6    1  Mm2
.......................................................Uuggacggagaacugauaag......................................  1    1  Mm2
.......................................................cuggacggagaacugauaag......................................  21   0  Mm2
.......................................................cuggacggagaacugauaaU......................................  1    1  Mm2
.......................................................cuggacggagaacugauaagg.....................................  71   0  Mm2
.......................................................cuggacggagaacugaCaagg.....................................  1    1  Mm2
.......................................................cuggacggagaacugauaagA.....................................  11   1  Mm2
.......................................................cuggacggagaacugauUagg.....................................  1    1  Mm2
```

Star **Mature**

gaauuuguccgaaagcggguccucgucauuuuuccgggccuguuuuuauacccuacuggacggagaacugauaagggcucgaauaggaccaagcaacauuugacguuaucugu			
....cuggacggagaacugauaagU.....................................	1	1	Mm2
....cuggacggagaacugauaaggg....................................	97	0	Mm2
....cuggacggagaacugauaaggA....................................	8	1	Mm2
....cuggacggagaacugauaaggU....................................	16	1	Mm2
....cuggacggagaacugUuaaggg....................................	1	1	Mm2
....cuggGcggagaacugauaaggg....................................	1	1	Mm2
....cuggacggagaacugauaaggC....................................	1	1	Mm2
....Auggacggagaacugauaagggc...................................	2	1	Mm2
....cugAacggagaacugauaagggc...................................	1	1	Mm2
....cuggacggagaacugauGagggc...................................	1	1	Mm2
....cuggacggagaacugauaGgggc...................................	1	1	Mm2
....cuggacggagaacugauaagggU...................................	5	1	Mm2
....cuggacggagaacugauaagggc...................................	233	0	Mm2
....cuggacggagaacugaCaagggc...................................	1	1	Mm2
....cuggacggagaacugauaagggA...................................	2	1	Mm2
....cAggacggagaacugauaagggc...................................	1	1	Mm2
....Uuggacggagaacugauaagggc...................................	1	1	Mm2
....cuggacggagaacugauaagggcC..................................	2	1	Mm2
....cuggCcggagaacugauaagggcu..................................	1	1	Mm2
....cuggacggagaacugauaagggcu..................................	18	0	Mm2
....uggacggagaacugauaa.......................................	1306	0	Mm2
....uggacggagaGcugauaa.......................................	1	1	Mm2
....uggacggagaacugauaU.......................................	39	1	Mm2
....uggGcggagaacugauaa.......................................	1	1	Mm2
....uggacggagaacugauUa.......................................	6	1	Mm2
....uggacggagaacugUuaa.......................................	1	1	Mm2
....uggacggGgaacugauaa.......................................	1	1	Mm2
....uggacgCagaacugauaa.......................................	1	1	Mm2
....uggacggagaacugaAaa.......................................	136	1	Mm2
....uggacggUgaacugauaa.......................................	2	1	Mm2
....uggacggagaacugauCa.......................................	1	1	Mm2
....uggUcggagaacugauaa.......................................	2	1	Mm2
....uggacggagaacugauaG.......................................	9	1	Mm2
....uggacggagaacugauaC.......................................	8	1	Mm2
....ugCacggagaacugauaa.......................................	2	1	Mm2
....uggacggagaacugauGa.......................................	2	1	Mm2
....uUgacggagaacugauaag......................................	1	1	Mm2
....uggacNgagaacugauaag......................................	1	1	Mm2
....uggacgCagaacugauaag......................................	1	1	Mm2
....uggacggagaacugauaaC......................................	11	1	Mm2
....uggacggagaacugauCag......................................	40	1	Mm2
....uggacggagaCcugauaag......................................	10	1	Mm2
....uggacggagaacugGuaag......................................	1	1	Mm2
....uggacggGgaacugauaag......................................	2	1	Mm2
....uggacggagUacugauaag......................................	2	1	Mm2
....uggacggagaacugUuaag......................................	1	1	Mm2
....ugAacggagaacugauaag......................................	2	1	Mm2
....ugCacggagaacugauaag......................................	1	1	Mm2
....uggacggagaGcugauaag......................................	20	1	Mm2
....uggGcggagaacugauaag......................................	1	1	Mm2
....uggacAgagaacugauaag......................................	1	1	Mm2
....uggacggagaacugauaCg......................................	33	1	Mm2
....uggaAggagaacugauaag......................................	1	1	Mm2
....uggacggagaacugaGaag......................................	1	1	Mm2
....uggacggagaacugauGag......................................	6	1	Mm2
....uggacggUgaacugauaag......................................	1	1	Mm2
....uggacggagaacCgauaag......................................	3	1	Mm2
....uggacggagGacugauaag......................................	12	1	Mm2
....uggacggagaacugauaGg......................................	24	1	Mm2
....uNgacggagaacugauaag......................................	1	1	Mm2
....uggacggagaacugaAaag......................................	2	1	Mm2
....Nggacggagaacugauaag......................................	4	1	Mm2
....uggaUggagaacugauaag......................................	1	1	Mm2
....uggacggagaacuAauaag......................................	2	1	Mm2
....uggacggagaacugaCaag......................................	1	1	Mm2
....uggacggagaacugauUag......................................	12	1	Mm2
....uggacggagaacugauaaU......................................	1753	1	Mm2
....uggacggagaacugauaaA......................................	3696	1	Mm2
....uCgacggagaacugauaag......................................	1	1	Mm2
....uggacgUagaacugauaag......................................	1	1	Mm2

```
                    Star                                  Mature
gaauuuguccgaaagcggguccucgucauuuuuccgggccuguuuuuauacccuacuggacggagaacugauaagggcucgaauaggaccaagcaacauuugacguuaucugu
........................................................Cggacggagaacugauaag......................................  2      1  Mm2
........................................................uggacggagaacugauaUg......................................  26     1  Mm2
........................................................uggacggagaacugauaag......................................  8357   0  Mm2
........................................................Aggacggagaacugauaag......................................  2      1  Mm2
........................................................uggacggaUaacugauaag......................................  2      1  Mm2
........................................................uggacggagaacuCauaag......................................  1      1  Mm2
........................................................uggacUgagaacugauaag......................................  3      1  Mm2
........................................................uggUcggagaacugauaag......................................  1      1  Mm2
........................................................uAgacggagaacugauaag......................................  4      1  Mm2
........................................................Gggacggagaacugauaagg.....................................  1      1  Mm2
........................................................uggacggUgaacugauaagg.....................................  4      1  Mm2
........................................................uggacggagaacugauaagA.....................................  2256   1  Mm2
........................................................uggacggagaacugauaagg.....................................  18110  0  Mm2
........................................................uggacgAagaacugauaagg.....................................  1      1  Mm2
........................................................uggacggagaacugGuaagg.....................................  2      1  Mm2
........................................................uggacggaAaacugauaagg.....................................  1      1  Mm2
........................................................ugUacggagaacugauaagg.....................................  6      1  Mm2
........................................................uggacggGgaacugauaagg.....................................  1      1  Mm2
........................................................uCgacggagaacugauaagg.....................................  5      1  Mm2
........................................................uggacggagGacugauaagg.....................................  24     1  Mm2
........................................................uggacUgagaacugauaagg.....................................  11     1  Mm2
........................................................uggacggagaacugUuaagg.....................................  1      1  Mm2
........................................................uggacggagaacugauaUgg.....................................  63     1  Mm2
........................................................uggacggagUacugauaagg.....................................  3      1  Mm2
........................................................uggacggagaacCgauaagg.....................................  2      1  Mm2
........................................................uggacggagaacugauaagC.....................................  10     1  Mm2
........................................................uggGcggagaacugauaagg.....................................  4      1  Mm2
........................................................uggacggaUaacugauaagg.....................................  4      1  Mm2
........................................................uggacgNagaacugauaagg.....................................  3      1  Mm2
........................................................uggacggagaacuCauaagg.....................................  4      1  Mm2
........................................................uggacggagaacugauaaUg.....................................  1      1  Mm2
........................................................uggacggagaacugauaGgg.....................................  90     1  Mm2
........................................................uggacgUagaacugauaagg.....................................  8      1  Mm2
........................................................uggacggagaacGgauaagg.....................................  1      1  Mm2
........................................................uggacNgagaacugauaagg.....................................  3      1  Mm2
........................................................uggUcggagaacugauaagg.....................................  1      1  Mm2
........................................................uggacggagaacuUauaagg.....................................  1      1  Mm2
........................................................uggacggagaacugauaaAg.....................................  116    1  Mm2
........................................................uUgacggagaacugauaagg.....................................  5      1  Mm2
........................................................uggaUggagaacugauaagg.....................................  1      1  Mm2
........................................................uggacggagaacugauGagg.....................................  16     1  Mm2
........................................................uggacggagaaUugauaagg.....................................  3      1  Mm2
........................................................uggCcggagaacugauaagg.....................................  3      1  Mm2
........................................................ugCacggagaacugauaagg.....................................  1      1  Mm2
........................................................uggacggagaacugauaagU.....................................  554    1  Mm2
........................................................uggacggagaacugauUagg.....................................  30     1  Mm2
........................................................uggacggagaacugauaCgg.....................................  71     1  Mm2
........................................................Nggacggagaacugauaagg.....................................  7      1  Mm2
........................................................uggacAgagaacugauaagg.....................................  4      1  Mm2
........................................................uggaGggagaacugauaagg.....................................  1      1  Mm2
........................................................Cggacggagaacugauaagg.....................................  4      1  Mm2
........................................................uggacggagaacuAauaagg.....................................  1      1  Mm2
........................................................uggaAggagaacugauaagg.....................................  2      1  Mm2
........................................................ugAacggagaacugauaagg.....................................  5      1  Mm2
........................................................uggacggagaacugauCagg.....................................  35     1  Mm2
........................................................uggacggagaacugaCaagg.....................................  3      1  Mm2
........................................................Aggacggagaacugauaagg.....................................  32     1  Mm2
........................................................uggacggagCacugauaagg.....................................  2      1  Mm2
........................................................uggacggagaCcugauaagg.....................................  60     1  Mm2
........................................................uAgacggagaacugauaagg.....................................  13     1  Mm2
........................................................uggacggagaGcugauaagg.....................................  103    1  Mm2
........................................................uggacggGgaacugauaaggg....................................  10     1  Mm2
........................................................uggCcggagaacugauaaggg....................................  3      1  Mm2
........................................................uggacggagGacugauaaggg....................................  50     1  Mm2
........................................................uggacggCgaacugauaaggg....................................  4      1  Mm2
........................................................Nggacggagaacugauaaggg....................................  22     1  Mm2
........................................................uggacggagaacugaCaaggg....................................  4      1  Mm2
........................................................uggacggUgaacugauaaggg....................................  8      1  Mm2
........................................................uggacggagaacugauaaCgg....................................  1      1  Mm2
........................................................uggacggaCaacugauaaggg....................................  4      1  Mm2
```

```
                    Star                                    Mature
gaauuuguccgaaagcggguccucgucauuuuuccgggccuguuuuuauacccuacuggacggagaacugauaagggcucgaauaggaccaagcaacauuugacguuaucugu
........................................................uggacggagaacCgauaaggg....................................    7      1    Mm2
........................................................uggacgNagaacugauaaggg....................................    4      1    Mm2
........................................................uggacggagaCcugauaaggg....................................    90     1    Mm2
........................................................uggacUgagaacugauaaggg....................................    14     1    Mm2
........................................................uggacggagaacugauaUggg....................................    129    1    Mm2
........................................................uggacNgagaacugauaaggg....................................    9      1    Mm2
........................................................uggacggagaacuUauaaggg....................................    11     1    Mm2
........................................................uggacggagaacuCauaaggg....................................    3      1    Mm2
........................................................uggacggagaacugauaCggg....................................    173    1    Mm2
........................................................Aggacggagaacugauaaggg....................................    41     1    Mm2
........................................................uUgacggagaacugauaaggg....................................    12     1    Mm2
........................................................uggacggagaacugauaaggg....................................    41055  0    Mm2
........................................................uggacggagaacugGuaaggg....................................    6      1    Mm2
........................................................uggacggagaacugauaaUgg....................................    5      1    Mm2
........................................................uggacgUagaacugauaaggg....................................    10     1    Mm2
........................................................uggacggagaacugauCaggg....................................    83     1    Mm2
........................................................uggacAgagaacugauaaggg....................................    12     1    Mm2
........................................................uggacggagaacugauaaggU....................................    899    1    Mm2
........................................................uggacCgagaacugauaaggg....................................    1      1    Mm2
........................................................uggacggagaaGugauaaggg....................................    1      1    Mm2
........................................................uggacggagaacugauaGggg....................................    262    1    Mm2
........................................................uggacggagaacugauaagAg....................................    15     1    Mm2
........................................................uggacggagaacugauaagCg....................................    3      1    Mm2
........................................................Cggacggagaacugauaaggg....................................    8      1    Mm2
........................................................uggUcggagaacugauaaggg....................................    2      1    Mm2
........................................................ugCacggagaacugauaaggg....................................    4      1    Mm2
........................................................uAgacggagaacugauaaggg....................................    11     1    Mm2
........................................................uggacggagaacugauGaggg....................................    49     1    Mm2
........................................................uggacggagaGcugauaaggg....................................    158    1    Mm2
........................................................uggacggagaacugUuaaggg....................................    6      1    Mm2
........................................................uggaGggagaacugauaaggg....................................    1      1    Mm2
........................................................uggacggagaaUugauaaggg....................................    16     1    Mm2
........................................................uggacggagaacugCuaaggg....................................    5      1    Mm2
........................................................uggacggagUacugauaaggg....................................    15     1    Mm2
........................................................uggGcggagaacugauaaggg....................................    7      1    Mm2
........................................................uggacggagaacGgauaaggg....................................    4      1    Mm2
........................................................uggacggagCacugauaaggg....................................    3      1    Mm2
........................................................uggacggagaacugauUaggg....................................    62     1    Mm2
........................................................uggaUggagaacugauaaggg....................................    9      1    Mm2
........................................................uggacggaAaacugauaaggg....................................    3      1    Mm2
........................................................uggacggaUaacugauaaggg....................................    8      1    Mm2
........................................................uggacggagaacugauaaggA....................................    5255   1    Mm2
........................................................uggacgCagaacugauaaggg....................................    5      1    Mm2
........................................................uggacgAagaacugauaaggg....................................    9      1    Mm2
........................................................uggacggagaacugauaaggC....................................    215    1    Mm2
........................................................uggacggagaacugauaagUg....................................    6      1    Mm2
........................................................uggaAggagaacugauaaggg....................................    3      1    Mm2
........................................................uggacggagaacuAauaaggg....................................    7      1    Mm2
........................................................ugAacggagaacugauaaggg....................................    14     1    Mm2
........................................................ugUacggagaacugauaaggg....................................    8      1    Mm2
........................................................uggacggagaaAugauaaggg....................................    3      1    Mm2
........................................................uggacggagaacugauaaAgg....................................    5      1    Mm2
........................................................Gggacggagaacugauaaggg....................................    1      1    Mm2
........................................................uAgacggagaacugauaagggc...................................    244    1    Mm2
........................................................uggCcggagaacugauaagggc...................................    116    1    Mm2
........................................................uggacggaAaacugauaagggc...................................    31     1    Mm2
........................................................uggacggagaacuUauaagggc...................................    45     1    Mm2
........................................................uggUcggagaacugauaagggc...................................    89     1    Mm2
........................................................uggacCgagaacugauaagggc...................................    13     1    Mm2
........................................................uggacgUagaacugauaagggc...................................    127    1    Mm2
........................................................uggacggagaacugauaaggAc...................................    162    1    Mm2
........................................................uggacggagaacugauaaggCc...................................    101    1    Mm2
........................................................uCgacggagaacugauaagggc...................................    37     1    Mm2
........................................................uggacggagaaUugauaagggc...................................    79     1    Mm2
........................................................uggacggagaacCgauaagggc...................................    74     1    Mm2
........................................................ugAacggagaacugauaagggc...................................    103    1    Mm2
........................................................uggacggagaacugauaagggA...................................    11213  1    Mm2
........................................................uggacgCagaacugauaagggc...................................    18     1    Mm2
........................................................uggacggagaacugaCaagggc...................................    45     1    Mm2
........................................................uggaGggagaacugauaagggc...................................    18     1    Mm2
```

```
                    Star                        Mature
gaauuuguccgaaagcggguccucgucauuuuuccgggccuguuuuuauacccuacuggacggagaacugauaagggcucgaauaggaccaagcaacauuugacguuaucugu
........................................................uggacggagaacugauaGgggc...................................	5049	1	Mm2
........................................................uggacggagaacugauUagggc...................................	638	1	Mm2
........................................................uggacggagaacuCauaagggc...................................	36	1	Mm2
........................................................uggacggagaacugUuaagggc...................................	68	1	Mm2
........................................................uggacggagaacugauaagAgc...................................	84	1	Mm2
........................................................uggacggagaacugauaUgggc...................................	1591	1	Mm2
........................................................uggacggUgaacugauaagggc...................................	113	1	Mm2
........................................................uggacggagaacugauaaCggc...................................	14	1	Mm2
........................................................uggacggagaacuAauaagggc...................................	105	1	Mm2
........................................................uggacggagaacugauaagUgc...................................	132	1	Mm2
........................................................uggaUggagaacugauaagggc...................................	99	1	Mm2
........................................................uggacggagaacugauaagCgc...................................	25	1	Mm2
........................................................uggacNgagaacugauaagggc...................................	79	1	Mm2
........................................................uggacgNagaacugauaagggc...................................	22	1	Mm2
........................................................ugCacggagaacugauaagggc...................................	46	1	Mm2
........................................................uggacggagUacugauaagggc...................................	112	1	Mm2
........................................................uggacggagaacAgauaagggc...................................	20	1	Mm2
........................................................uggGcggagaacugauaagggc...................................	131	1	Mm2
........................................................uNgacggagaacugauaagggc...................................	7	1	Mm2
........................................................uggacgAagaacugauaagggc...................................	63	1	Mm2
........................................................uggacggagaacugGuaagggc...................................	51	1	Mm2
........................................................uggacggagaacugauaagggU...................................	3929	1	Mm2
........................................................uggacggagaacugauaagggc...................................	505969	0	Mm2
........................................................uggacggagaacugaGaagggc...................................	8	1	Mm2
........................................................uggacggagaacugauaNgggc...................................	1	1	Mm2
........................................................uUgacggagaacugauaagggc...................................	92	1	Mm2
........................................................uggacggagaacugauCagggc...................................	1033	1	Mm2
........................................................uggacggCgaacugauaagggc...................................	23	1	Mm2
........................................................uggacggagaaAugauaagggc...................................	14	1	Mm2
........................................................uggacggagCacugauaagggc...................................	55	1	Mm2
........................................................uggacggagaCcugauaagggc...................................	435	1	Mm2
........................................................uggacggagaacugauaaUggc...................................	76	1	Mm2
........................................................Gggacggagaacugauaagggc...................................	27	1	Mm2
........................................................uggacggagaacugCuaagggc...................................	17	1	Mm2
........................................................uggacUgagaacugauaagggc...................................	84	1	Mm2
........................................................uggacggagaacugauaagggG...................................	327	1	Mm2
........................................................uggacggagaacugauaaggUc...................................	123	1	Mm2
........................................................uggacggagaacugauaaAggc...................................	46	1	Mm2
........................................................uggacggagaGcugauaagggc...................................	844	1	Mm2
........................................................uggaAggagaacugauaagggc...................................	29	1	Mm2
........................................................uggacggagaaGugauaagggc...................................	14	1	Mm2
........................................................uggacggagGacugauaagggc...................................	998	1	Mm2
........................................................uggacggaUaacugauaagggc...................................	93	1	Mm2
........................................................uggacggagaacugauaCgggc...................................	1933	1	Mm2
........................................................uggacggagaacugaAaagggc...................................	14	1	Mm2
........................................................ugUacggagaacugauaagggc...................................	92	1	Mm2
........................................................uggacggagaacGgauaagggc...................................	22	1	Mm2
........................................................Nggacggagaacugauaagggc...................................	258	1	Mm2
........................................................Cggacggagaacugauaagggc...................................	64	1	Mm2
........................................................uggacggGgaacugauaagggc...................................	113	1	Mm2
........................................................ugNacggagaacugauaagggc...................................	5	1	Mm2
........................................................uggacAgagaacugauaagggc...................................	82	1	Mm2
........................................................uggacggagaacugauGagggc...................................	745	1	Mm2
........................................................Aggacggagaacugauaagggc...................................	129	1	Mm2
........................................................uggacggaCaacugauaagggc...................................	27	1	Mm2
........................................................uggacggUgaacugauaagggcu..................................	6	1	Mm2
........................................................uggacggagaacugauaagggGu..................................	10	1	Mm2
........................................................uCgacggagaacugauaagggcu..................................	2	1	Mm2
........................................................uggacggagGacugauaagggcu..................................	45	1	Mm2
........................................................uggacggagaacugauaGgggcu..................................	340	1	Mm2
........................................................uAgacggagaacugauaagggcu..................................	10	1	Mm2
........................................................uggacggagaacugUuaagggcu..................................	10	1	Mm2
........................................................uggacggagaacugauaagggUu..................................	632	1	Mm2
........................................................uggacggagaacugaCaagggcu..................................	3	1	Mm2
........................................................uggacggagaCcugauaagggcu..................................	13	1	Mm2
........................................................uggacgNagaacugauaagggcu..................................	1	1	Mm2
........................................................uggacggagCacugauaagggcu..................................	6	1	Mm2
........................................................uggacggagaacugauaagggcC..................................	305	1	Mm2
........................................................uggacggagaacugauaagggAu..................................	672	1	Mm2
........................................................uggacggagaacugauaagggcG..................................	1203	1	Mm2
```

Star **Mature**

```
gaauuuguccgaaagcggguccucgucauuuuuccgggccuguuuuuauacccuacuggacggagaacugauaagggcucgaauaggaccaagcaacauuugacguuaucugu
........................................................uggacggagaacugauaaggUcu..................................    8      1   Mm2
........................................................uggacggagaGcugauaagggcu..................................    37     1   Mm2
........................................................uggUcggagaacugauaagggcu..................................    1      1   Mm2
........................................................ugUacggagaacugauaagggcu..................................    5      1   Mm2
........................................................uggacggaAaacugauaagggcu..................................    2      1   Mm2
........................................................uggacCgagaacugauaagggcu..................................    2      1   Mm2
........................................................uggacgUagaacugauaagggcu..................................    7      1   Mm2
........................................................uggacggagaacugauaCgggcu..................................    93     1   Mm2
........................................................uggacggagaacugauaaggAcu..................................    5      1   Mm2
........................................................uggacggagaacuAauaagggcu..................................    3      1   Mm2
........................................................uggacggagaacuCauaagggcu..................................    2      1   Mm2
........................................................ugCacggagaacugauaagggcu..................................    2      1   Mm2
........................................................uggaGggagaacugauaagggcu..................................    2      1   Mm2
........................................................Nggacggagaacugauaagggcu..................................    10     1   Mm2
........................................................uggacAgagaacugauaagggcu..................................    3      1   Mm2
........................................................uggCcggagaacugauaagggcu..................................    6      1   Mm2
........................................................uggacggagaacugauUagggcu..................................    27     1   Mm2
........................................................uggacggagaacugauaUgggcu..................................    74     1   Mm2
........................................................uggacggagUacugauaagggcu..................................    8      1   Mm2
........................................................uggacggagaacugCuaagggcu..................................    1      1   Mm2
........................................................uggacggagaacugGuaagggcu..................................    5      1   Mm2
........................................................uggacggagaacugauCagggcu..................................    47     1   Mm2
........................................................uggacggaCaacugauaagggcu..................................    1      1   Mm2
........................................................uggacggagaaGugauaagggcu..................................    2      1   Mm2
........................................................uggacggagaacugauGagggcu..................................    36     1   Mm2
........................................................uggacggaUaacugauaagggcu..................................    3      1   Mm2
........................................................uggacggagaacugauaaggCcu..................................    2      1   Mm2
........................................................uggacggagaacuUauaagggcu..................................    3      1   Mm2
........................................................uggacgAagaacugauaagggcu..................................    4      1   Mm2
........................................................uggaAggagaacugauaagggcu..................................    3      1   Mm2
........................................................uggacggagaaUugauaagggcu..................................    3      1   Mm2
........................................................uUgacggagaacugauaagggcu..................................    7      1   Mm2
........................................................Cggacggagaacugauaagggcu..................................    5      1   Mm2
........................................................uggGcggagaacugauaagggcu..................................    8      1   Mm2
........................................................uggacNgagaacugauaagggcu..................................    2      1   Mm2
........................................................uggaUggagaacugauaagggcu..................................    8      1   Mm2
........................................................Aggacggagaacugauaagggcu..................................    8      1   Mm2
........................................................uggacgCagaacugauaagggcu..................................    2      1   Mm2
........................................................uggacUgagaacugauaagggcu..................................    5      1   Mm2
........................................................uggacggagaacugauaagggcu..................................    25196  0   Mm2
........................................................uggacggagaacCgauaagggcu..................................    2      1   Mm2
........................................................uggacggagaacugauaaUggcu..................................    3      1   Mm2
........................................................uggacggagaacugauaaCggcu..................................    4      1   Mm2
........................................................uggacggagaacAgauaagggcu..................................    2      1   Mm2
........................................................uggacggagaacGgauaagggcu..................................    3      1   Mm2
........................................................ugAacggagaacugauaagggcu..................................    4      1   Mm2
........................................................uggacggGgaacugauaagggcu..................................    9      1   Mm2
........................................................uggacggagaacugauaagggcCc.................................    1      1   Mm2
........................................................uggacggagaacugauaGgggcuc.................................    1      1   Mm2
........................................................uggacggagaacugauaagggcuG.................................    51     1   Mm2
........................................................uggacggagaUcugauaagggcuc.................................    9      1   Mm2
........................................................uggacggagaacugauaagggcAc.................................    27     1   Mm2
........................................................uggacggagaacugauaagggcuA.................................    123    1   Mm2
........................................................uggacggagaacugauaagggcuc.................................    73     0   Mm2
........................................................uggacggagaacugauaCgggcuc.................................    1      1   Mm2
........................................................uggacggagaacugauaagggcuGg................................    1      1   Mm2
........................................................uggacggagaacugauaagggcucU................................    2      1   Mm2
.........................................................ggacggagaacugauaag......................................    17     0   Mm2
.........................................................ggacggagaaAugauaag......................................    1      1   Mm2
.........................................................ggacggagaacugauaaU......................................    1      1   Mm2
.........................................................ggacggagaacugauaaA......................................    8      1   Mm2
.........................................................ggGcggagaacugauaagg.....................................    1      1   Mm2
.........................................................ggacggagaacugauaagA.....................................    5      1   Mm2
.........................................................ggacggagaacuUauaagg.....................................    1      1   Mm2
.........................................................Ugacggagaacugauaagg.....................................    2      1   Mm2
.........................................................ggacggagaacugauaagU.....................................    1      1   Mm2
.........................................................ggacggagaGcugauaagg.....................................    1      1   Mm2
.........................................................ggacggagaacugauaUgg.....................................    1      1   Mm2
.........................................................ggacggagaacugauaagg.....................................    47     0   Mm2
.........................................................Ugacggagaacugauaaggg....................................    1      1   Mm2
```

Star **Mature**

```
gaauuuguccgaaagcggguccucgucauuuuuccgggccuguuuuuauacccuacuggacggagaacugauaagggcucgaauaggaccaagcaacauuugacguuaucugu
.........................................................ggacggaAaacugauaaggg....................................  1    1  Mm2
.........................................................ggacggagaacugauaaggU....................................  3    1  Mm2
.........................................................ggacggagaaAugauaaggg....................................  2    1  Mm2
.........................................................ggacggagaacugauaaggA....................................  11   1  Mm2
.........................................................ggacggagaacugaCaaggg....................................  1    1  Mm2
.........................................................ggacggagaacugauaaggC....................................  2    1  Mm2
.........................................................ggacggagUacugauaaggg....................................  1    1  Mm2
.........................................................ggacggagaacugauaaggg....................................  48   0  Mm2
.........................................................ggacggGgaacugauaagggc...................................  1    1  Mm2
.........................................................ggacggagaacugauaagggA...................................  26   1  Mm2
.........................................................ggacggagaacugauaaggUc...................................  3    1  Mm2
.........................................................ggacggagaGcugauaagggc...................................  1    1  Mm2
.........................................................ggacggagaacugauaUgggc...................................  2    1  Mm2
.........................................................ggacggagaacugauaagCgc...................................  2    1  Mm2
.........................................................ggacCgagaacugauaagggc...................................  1    1  Mm2
.........................................................ggacggagaaUugauaagggc...................................  3    1  Mm2
.........................................................ggacggagaacugauaagggc...................................  887  0  Mm2
.........................................................ggacggagaaGugauaagggc...................................  4    1  Mm2
.........................................................ggacggagUacugauaagggc...................................  1    1  Mm2
.........................................................ggGcggagaacugauaagggc...................................  2    1  Mm2
.........................................................ggacggagaacugauaaggAc...................................  2    1  Mm2
.........................................................ggacggagaacugauaagggU...................................  9    1  Mm2
.........................................................ggacggagaaAugauaagggc...................................  2    1  Mm2
.........................................................ggacggagaCcugauaagggc...................................  1    1  Mm2
.........................................................ggacggagaacugauaaggCc...................................  5    1  Mm2
.........................................................ggacggagaacugauaGgggc...................................  12   1  Mm2
.........................................................ggacggagaacugauaagUgc...................................  1    1  Mm2
.........................................................ggacggagaacugauaagAgc...................................  1    1  Mm2
.........................................................ggaAggagaacugauaagggc...................................  1    1  Mm2
.........................................................Ugacggagaacugauaagggc...................................  20   1  Mm2
.........................................................ggacggagaacuUauaagggc...................................  2    1  Mm2
.........................................................ggUcggagaacugauaagggc...................................  2    1  Mm2
.........................................................ggacgAagaacugauaagggc...................................  1    1  Mm2
.........................................................ggacggagCacugauaagggc...................................  8    1  Mm2
.........................................................ggacggagaacugauaaUggc...................................  1    1  Mm2
.........................................................ggacgUagaacugauaagggc...................................  1    1  Mm2
.........................................................ggacggaAaacugauaagggc...................................  1    1  Mm2
.........................................................ggacggagaacugauCagggc...................................  3    1  Mm2
.........................................................gAacggagaacugauaagggc...................................  2    1  Mm2
.........................................................ggacggagaacugauaCgggc...................................  4    1  Mm2
.........................................................ggaGggagaacugauaagggc...................................  1    1  Mm2
.........................................................ggacggagaacugauaagggcG..................................  3    1  Mm2
.........................................................ggacggagaacugauaagggcu..................................  33   0  Mm2
.........................................................ggacggagaacugauaaggUcu..................................  1    1  Mm2
.........................................................ggacggagGacugauaagggcu..................................  2    1  Mm2
.........................................................ggacggagaacugauaagggcC..................................  1    1  Mm2
.........................................................Ugacggagaacugauaagggcu..................................  1    1  Mm2
.........................................................ggacCgagaacugauaagggcu..................................  1    1  Mm2
.........................................................ggacggagaacugauaagggAu..................................  1    1  Mm2
.........................................................ggacggagaacugauaaCggcu..................................  1    1  Mm2
.........................................................ggacggagaacugauaagggUu..................................  1    1  Mm2
.........................................................ggacggagaacugauaaUggcu..................................  1    1  Mm2
..........................................................gacggagaacugauaagggc...................................  1    0  Mm2
...........................................................acggagaacugauaaggg....................................  1    0  Mm2
...........................................................acggagaacugauaagggc...................................  3    0  Mm2
...........................................................acUgagaacugauaagggc...................................  1    1  Mm2
............................................................cggagaacugauaagggc...................................  1    0  Mm2
............................................................Uggagaacugauaagggc...................................  3    1  Mm2

....................ccucgucauuuuuccgggccugu......................................................................  13   0  Mm1
....................ccucgucauuuuuccgUgccugu......................................................................  1    1  Mm1
......................................................Ucuggacggagaacugauaagggc...................................  1    1  Mm1
.......................................................cuggacggagaacugauaag......................................  1    0  Mm1
.......................................................cuggacggagaacugauaagA.....................................  7    1  Mm1
.......................................................cuggacggagaacugauaagg.....................................  43   0  Mm1
.......................................................cuggacggagCacugauaagg.....................................  1    1  Mm1
.......................................................cuggacggagCacugauaaggg....................................  1    1  Mm1
.......................................................cuggacggagaacugauaaggg....................................  17   0  Mm1
.......................................................cuggacggagCacugauaagggc...................................  1    1  Mm1
.......................................................cugUacggagaacugauaagggc...................................  1    1  Mm1
```

Star **Mature**

gaauuuguccgaaagcgggucc ucgucauuuuuccgggccuguuuuuauacccuacuggacggagaacugauaagggcucgaauaggaccaagcaacauuugacguuaucugu			
....cuggacggagaacugauaagggc...................................	45	0	Mm1
....uggacggagaacugaCaa.......................................	1	1	Mm1
....uAgacggagaacugauaa.......................................	2	1	Mm1
....uggacggagaacugauaU.......................................	29	1	Mm1
....uggacUgagaacugauaa.......................................	1	1	Mm1
....uggacggagaacugaAaa.......................................	64	1	Mm1
....uggacggagaGcugauaa.......................................	1	1	Mm1
....uggacgUagaacugauaa.......................................	1	1	Mm1
....ugAacggagaacugauaa.......................................	1	1	Mm1
....uggacggagaacugauaa.......................................	995	0	Mm1
....uggacggagaacuAauaa.......................................	1	1	Mm1
....uggacggagaacugauaC.......................................	2	1	Mm1
....Nggacggagaacugauaa.......................................	2	1	Mm1
....uggacggagaacugauaG.......................................	2	1	Mm1
....uggacggagaacugauCa.......................................	3	1	Mm1
....uggacgAagaacugauaa.......................................	1	1	Mm1
....Aggacggagaacugauaa.......................................	1	1	Mm1
....uggacggagaacugauUa.......................................	6	1	Mm1
....uggacggagaacugauGa.......................................	1	1	Mm1
....uggacgAagaacugauaag......................................	1	1	Mm1
....Aggacggagaacugauaag......................................	3	1	Mm1
....uggacggagaGcugauaag......................................	19	1	Mm1
....uggacggagaacugauGag......................................	3	1	Mm1
....Nggacggagaacugauaag......................................	3	1	Mm1
....uggacggagaacugauaCg......................................	16	1	Mm1
....uggacggagaacugauaaA......................................	2940	1	Mm1
....uggacggagaacugGuaag......................................	1	1	Mm1
....ugCacggagaacugauaag......................................	1	1	Mm1
....uggacggagaacugauaaU......................................	1409	1	Mm1
....uggacggagaacugauaag......................................	3971	0	Mm1
....uggacggagaCcugauaag......................................	7	1	Mm1
....uggacggagGacugauaag......................................	6	1	Mm1
....uggacggagaacugauCag......................................	2	1	Mm1
....uggacggagUacugauaag......................................	2	1	Mm1
....uggaUggagaacugauaag......................................	1	1	Mm1
....uggacggagaacugUuaag......................................	2	1	Mm1
....uggacgCagaacugauaag......................................	1	1	Mm1
....uggUcggagaacugauaag......................................	1	1	Mm1
....ugAacggagaacugauaag......................................	1	1	Mm1
....uggacggagaacugauaaC......................................	20	1	Mm1
....uAgacggagaacugauaag......................................	2	1	Mm1
....uggacggaAaacugauaag......................................	2	1	Mm1
....uggacAgagaacugauaag......................................	2	1	Mm1
....uggacggagaacugauaUg......................................	14	1	Mm1
....uggacggagaacugauUag......................................	5	1	Mm1
....uggacggagaacugauaGg......................................	15	1	Mm1
....uCgacggagaacugauaagg.....................................	2	1	Mm1
....uggacggUgaacugauaagg.....................................	6	1	Mm1
....uggacAgagaacugauaagg.....................................	5	1	Mm1
....uggacCgagaacugauaagg.....................................	2	1	Mm1
....uggaAggagaacugauaagg.....................................	2	1	Mm1
....uggacggagaacugauaUgg.....................................	70	1	Mm1
....uggacggagaacCgauaagg.....................................	4	1	Mm1
....uggacggagaCcugauaagg.....................................	70	1	Mm1
....uggacggagaacugauCagg.....................................	29	1	Mm1
....Aggacggagaacugauaagg.....................................	53	1	Mm1
....uggacggGgaacugauaagg.....................................	1	1	Mm1
....uggacUgagaacugauaagg.....................................	3	1	Mm1
....Nggacggagaacugauaagg.....................................	6	1	Mm1
....uggacggagaacAgauaagg.....................................	2	1	Mm1
....uggacggCgaacugauaagg.....................................	1	1	Mm1
....uAgacggagaacugauaagg.....................................	5	1	Mm1
....uggUcggagaacugauaagg.....................................	6	1	Mm1
....uggacggagaacuCauaagg.....................................	2	1	Mm1
....uggacNgagaacugauaagg.....................................	3	1	Mm1
....uggacggagaacugauaaggU....................................	365	1	Mm1
....uggGcggagaacugauaagg.....................................	4	1	Mm1
....uggacggagaacugUuaagg.....................................	2	1	Mm1
....uggacggagaacugaGaagg.....................................	2	1	Mm1
....ugUacggagaacugauaagg.....................................	3	1	Mm1

Star Mature

gaauuuguccgaaagcggguccucgucauuuuuccgggccuguuuuuauacccuacuggacggagaacugauaagggcucgaauaggaccaagcaacauuugacguuaucugu			
....uggacggagaacugauUagg.....................................	29	1	Mm1
....uggacggagaacugaCaagg.....................................	1	1	Mm1
....uggacggagaacugauaaAg.....................................	24	1	Mm1
....uggacggagUacugauaagg.....................................	6	1	Mm1
....uggacggagaacugauGagg.....................................	40	1	Mm1
....uggacgAagaacugauaagg.....................................	1	1	Mm1
....uggacggagaacugauaagC.....................................	3	1	Mm1
....uggacggagaacugaAaagg.....................................	1	1	Mm1
....uggacggagaacugauaaUg.....................................	1	1	Mm1
....uggacggaUaacugauaagg.....................................	1	1	Mm1
....uggacggagaacugauaagA.....................................	1788	1	Mm1
....uggacgCagaacugauaagg.....................................	1	1	Mm1
....uUgacggagaacugauaagg.....................................	5	1	Mm1
....uggacgUagaacugauaagg.....................................	7	1	Mm1
....uggacggagaacugGuaagg.....................................	3	1	Mm1
....uggacggagaaGugauaagg.....................................	1	1	Mm1
....uggacggagaacugauaagg.....................................	17362	0	Mm1
....uggacggagaacuUauaagg.....................................	1	1	Mm1
....uggacggagaGcugauaagg.....................................	122	1	Mm1
....uggacggagaaUugauaagg.....................................	14	1	Mm1
....ugAacggagaacugauaagg.....................................	1	1	Mm1
....uggaUggagaacugauaagg.....................................	3	1	Mm1
....ugCacggagaacugauaagg.....................................	2	1	Mm1
....uggacggagaacugauaCgg.....................................	71	1	Mm1
....uggacggagGacugauaagg.....................................	8	1	Mm1
....Cggacggagaacugauaagg.....................................	3	1	Mm1
....uggacggagCacugauaagg.....................................	5	1	Mm1
....uggacggagaacugauaGgg.....................................	77	1	Mm1
....uggaGggagaacugauaagg.....................................	1	1	Mm1
....uggacggagaacugGuaaggg....................................	5	1	Mm1
....uggacggaUaacugauaaggg....................................	1	1	Mm1
....uggaUggagaacugauaaggg....................................	1	1	Mm1
....uggacggagaacugauaaggg....................................	14223	0	Mm1
....uggacUgagaacugauaaggg....................................	3	1	Mm1
....uggacggagaacugauUaggg....................................	24	1	Mm1
....uggacAgagaacugauaaggg....................................	3	1	Mm1
....uggacggagaacAgauaaggg....................................	1	1	Mm1
....uggacggagaGcugauaaggg....................................	85	1	Mm1
....uggacNgagaacugauaaggg....................................	1	1	Mm1
....uggacggagaacugauaagCg....................................	1	1	Mm1
....uggacggagaacugauCaggg....................................	29	1	Mm1
....Nggacggagaacugauaaggg....................................	6	1	Mm1
....uggGcggagaacugauaaggg....................................	3	1	Mm1
....uggacggagGacugauaaggg....................................	3	1	Mm1
....uggacggagCacugauaaggg....................................	2	1	Mm1
....uggacggagaacugauaaggU....................................	413	1	Mm1
....ugUacggagaacugauaaggg....................................	1	1	Mm1
....uggacgCagaacugauaaggg....................................	1	1	Mm1
....uggacggagaaAugauaaggg....................................	1	1	Mm1
....uggacggagaacCgauaaggg....................................	2	1	Mm1
....Cggacggagaacugauaaggg....................................	2	1	Mm1
....uggacgUagaacugauaaggg....................................	2	1	Mm1
....uAgacggagaacugauaaggg....................................	22	1	Mm1
....uggacggagaacugauaUggg....................................	41	1	Mm1
....uggacggUgaacugauaaggg....................................	4	1	Mm1
....uggacggagaacugauaCggg....................................	59	1	Mm1
....uggacggagaacugauGaggg....................................	18	1	Mm1
....uggacggGgaacugauaaggg....................................	2	1	Mm1
....uggacggagaacugauaaUgg....................................	3	1	Mm1
....uggacggagaacugauaaggA....................................	3716	1	Mm1
....uUgacggagaacugauaaggg....................................	1	1	Mm1
....uggacggagaCcugauaaggg....................................	33	1	Mm1
....uggacggCgaacugauaaggg....................................	1	1	Mm1
....uggUcggagaacugauaaggg....................................	3	1	Mm1
....uggacggagaacugauaGggg....................................	82	1	Mm1
....uCgacggagaacugauaaggg....................................	3	1	Mm1
....uggacggagaacuAauaaggg....................................	3	1	Mm1
....uggacggagaacugUuaaggg....................................	4	1	Mm1
....uggacggagaacugauaagUg....................................	3	1	Mm1
....uggacggagUacugauaaggg....................................	7	1	Mm1

```
                    Star                                Mature
gaauuuguccgaaagcggguccucgucauuuuuccgggccuguuuuuauacccuacuggacggagaacugauaagggcucgaauaggaccaagcaacauuugacguuaucugu
........................................................ugAacggagaacugauaaggg....................................   3      1   Mm1
........................................................uggacggagaacugauaaggC....................................   5      1   Mm1
........................................................uggacggagaaUugauaaggg....................................   1      1   Mm1
........................................................Aggacggagaacugauaaggg....................................   31     1   Mm1
........................................................uggaGggagaacugauaagggc...................................   1      1   Mm1
........................................................uAgacggagaacugauaagggc...................................   47     1   Mm1
........................................................uggacggagaacugGuaagggc...................................   9      1   Mm1
........................................................uggacggagaacugauaaggUc...................................   26     1   Mm1
........................................................uggacNgagaacugauaagggc...................................   11     1   Mm1
........................................................uggacggaCaacugauaagggc...................................   6      1   Mm1
........................................................uggacggagaacugauaaCggc...................................   2      1   Mm1
........................................................uggaAggagaacugauaagggc...................................   8      1   Mm1
........................................................uggacggagaacugauaagggA...................................   4855   1   Mm1
........................................................uggacggagaacugauGagggc...................................   154    1   Mm1
........................................................uggacggagaacugaCaagggc...................................   11     1   Mm1
........................................................uggacAgagaacugauaagggc...................................   10     1   Mm1
........................................................ugUacggagaacugauaagggc...................................   10     1   Mm1
........................................................uCgacggagaacugauaagggc...................................   10     1   Mm1
........................................................uggacggagUacugauaagggc...................................   49     1   Mm1
........................................................uggacggagaaAugauaagggc...................................   6      1   Mm1
........................................................uggacggagaacugauaagAgc...................................   24     1   Mm1
........................................................uggacggagaacuAauaagggc...................................   18     1   Mm1
........................................................uggacggagaacugauaUgggc...................................   321    1   Mm1
........................................................uggacgUagaacugauaagggc...................................   17     1   Mm1
........................................................uggacggagaacGgauaagggc...................................   3      1   Mm1
........................................................uggacggagGacugauaagggc...................................   251    1   Mm1
........................................................uggUcggagaacugauaagggc...................................   13     1   Mm1
........................................................uUgacggagaacugauaagggc...................................   18     1   Mm1
........................................................uggacggagaacugauaaAggc...................................   21     1   Mm1
........................................................uggCcggagaacugauaagggc...................................   24     1   Mm1
........................................................uggacggagaacugUuaagggc...................................   9      1   Mm1
........................................................Nggacggagaacugauaagggc...................................   48     1   Mm1
........................................................uggacggagaacugauaagCgc...................................   8      1   Mm1
........................................................uggacggagaCcugauaagggc...................................   88     1   Mm1
........................................................uggacggagaacugauaaggCc...................................   6      1   Mm1
........................................................uggacggagaacugauaagggU...................................   821    1   Mm1
........................................................uggacggagaacugauaagUgc...................................   36     1   Mm1
........................................................uggacggagaacugauCagggc...................................   209    1   Mm1
........................................................uggacggagaacugauaaUggc...................................   17     1   Mm1
........................................................uggacggagaacCgauaagggc...................................   10     1   Mm1
........................................................uggacggagaacugauUagggc...................................   148    1   Mm1
........................................................uggacggGgaacugauaagggc...................................   32     1   Mm1
........................................................uggacggaAaacugauaagggc...................................   6      1   Mm1
........................................................uggacggagaacugauNagggc...................................   1      1   Mm1
........................................................Aggacggagaacugauaagggc...................................   53     1   Mm1
........................................................uggacggagaacuCauaagggc...................................   12     1   Mm1
........................................................uggacggaUaacugauaagggc...................................   19     1   Mm1
........................................................uggacggagaacugauaGgggc...................................   762    1   Mm1
........................................................uggacggagaacugaAaagggc...................................   3      1   Mm1
........................................................uggacggagaaGugauaagggc...................................   2      1   Mm1
........................................................uNgacggagaacugauaagggc...................................   1      1   Mm1
........................................................uggacgCagaacugauaagggc...................................   7      1   Mm1
........................................................uggacggagaacuUauaagggc...................................   8      1   Mm1
........................................................uggacggagCacugauaagggc...................................   27     1   Mm1
........................................................uggaUggagaacugauaagggc...................................   20     1   Mm1
........................................................uggacggagaacAgauaagggc...................................   5      1   Mm1
........................................................uggacggagaacugCuaagggc...................................   3      1   Mm1
........................................................uggacggagaacugauaCgggc...................................   394    1   Mm1
........................................................ugCacggagaacugauaagggc...................................   8      1   Mm1
........................................................uggacggagaaUugauaagggc...................................   20     1   Mm1
........................................................uggacgNagaacugauaagggc...................................   3      1   Mm1
........................................................uggacggagaacugauaaggGG...................................   16     1   Mm1
........................................................uggacggagaacugauaagggc...................................   99594  0   Mm1
........................................................ugAacggagaacugauaagggc...................................   19     1   Mm1
........................................................uggacggagaGcugauaagggc...................................   209    1   Mm1
........................................................uggGcggagaacugauaagggc...................................   25     1   Mm1
........................................................uggacUgagaacugauaagggc...................................   17     1   Mm1
........................................................uggacgAagaacugauaagggc...................................   8      1   Mm1
........................................................uggacggCgaacugauaagggc...................................   3      1   Mm1
........................................................uggacggUgaacugauaagggc...................................   47     1   Mm1
```

Star Mature

```
gaauuuguccgaaagcggguccucgucauuuuuccgggccuguuuuuauacccuacuggacggagaacugauaagggcucgaauaggaccaagcaacauuugacguuaucugu
........................................................Cggacggagaacugauaagggc...................................  9     1  Mm1
........................................................uggacggagaacugauaaggAc...................................  18    1  Mm1
........................................................uggacggagaacugauaaAggcu..................................  1     1  Mm1
........................................................uggacggaUaacugauaagggcu..................................  1     1  Mm1
........................................................uggacggagaacugauaagUgcu..................................  1     1  Mm1
........................................................uUgacggagaacugauaagggcu..................................  2     1  Mm1
........................................................uggacggagaacugauaagggGu..................................  10    1  Mm1
........................................................uggacggagaacugaCaagggcu..................................  1     1  Mm1
........................................................uggacgUagaacugauaagggcu..................................  3     1  Mm1
........................................................uggacggagaCcugauaagggcu..................................  9     1  Mm1
........................................................uggacAgagaacugauaagggcu..................................  1     1  Mm1
........................................................uggacggagaacugUuaagggcu..................................  3     1  Mm1
........................................................uggacggagaacugauGagggcu..................................  4     1  Mm1
........................................................uggacggagaacuAauaagggcu..................................  1     1  Mm1
........................................................ugCacggagaacugauaagggcu..................................  2     1  Mm1
........................................................uggacggagaacugauaaggUcu..................................  3     1  Mm1
........................................................uggacggagUacugauaagggcu..................................  3     1  Mm1
........................................................uggUcggagaacugauaagggcu..................................  1     1  Mm1
........................................................uggacggagaacugauaagggcG..................................  66    1  Mm1
........................................................uggacNgagaacugauaagggcu..................................  2     1  Mm1
........................................................uggacggagaacugGuaagggcu..................................  2     1  Mm1
........................................................uggacggagaacugauCagggcu..................................  12    1  Mm1
........................................................uggacggagaaGugauaagggcu..................................  1     1  Mm1
........................................................uggaUggagaacugauaagggcu..................................  1     1  Mm1
........................................................uggacggagaGcugauaagggcu..................................  14    1  Mm1
........................................................uggacggagaacugauUagggcu..................................  11    1  Mm1
........................................................uggacggagaacugauaagggcu..................................  4894  0  Mm1
........................................................uAgacggagaacugauaagggcu..................................  2     1  Mm1
........................................................Aggacggagaacugauaagggcu..................................  2     1  Mm1
........................................................uggacggagaacugauaaUggcu..................................  1     1  Mm1
........................................................uggacggagaacugauaUgggcu..................................  23    1  Mm1
........................................................uggacggUgaacugauaagggcu..................................  1     1  Mm1
........................................................uggacggagaacugauaagggUu..................................  147   1  Mm1
........................................................uggacggagaacugauaGgggcu..................................  26    1  Mm1
........................................................uggacggagaacugauaagggcC..................................  44    1  Mm1
........................................................uggacggagaacugauaagCgcu..................................  1     1  Mm1
........................................................uggCcggagaacugauaagggcu..................................  1     1  Mm1
........................................................uggacggagGacugauaagggcu..................................  14    1  Mm1
........................................................uggacggagaacugauaCgggcu..................................  21    1  Mm1
........................................................uggacUgagaacugauaagggcu..................................  1     1  Mm1
........................................................uggacggagaacugauaagggAu..................................  284   1  Mm1
........................................................uggacggagaacugauaagggcuA.................................  18    1  Mm1
........................................................uggacggagaUcugauaagggcuc.................................  5     1  Mm1
........................................................uggacggagaacugauaagggcAc.................................  14    1  Mm1
........................................................uggacggagaacugauaagggcuc.................................  22    0  Mm1
........................................................uggacggagaacugauaagggcuGgaa..............................  1     1  Mm1
.........................................................ggacggagaacugauaaA......................................  9     1  Mm1
.........................................................ggacggagaaAugauaag......................................  1     1  Mm1
.........................................................ggacggagaacugauaag......................................  9     0  Mm1
.........................................................ggacggagaacugauaagg.....................................  27    0  Mm1
.........................................................ggacggagaacugauaagU.....................................  5     1  Mm1
.........................................................ggacggagCacugauaagg.....................................  1     1  Mm1
.........................................................ggGcggagaacugauaaggg....................................  1     1  Mm1
.........................................................ggacggagaacugauaaggg....................................  43    0  Mm1
.........................................................Ugacggagaacugauaaggg....................................  1     1  Mm1
.........................................................ggacggagaacugauaaggC....................................  1     1  Mm1
.........................................................ggacggagaacugauaaggA....................................  7     1  Mm1
.........................................................ggacggagaacugauaaggAc...................................  1     1  Mm1
.........................................................ggacggagGacugauaagggc...................................  2     1  Mm1
.........................................................ggGcggagaacugauaagggc...................................  2     1  Mm1
.........................................................ggacggagaacugauaaUggc...................................  1     1  Mm1
.........................................................ggacggagaCcugauaagggc...................................  1     1  Mm1
.........................................................ggacggaAaacugauaagggc...................................  1     1  Mm1
.........................................................ggacggagaacugauaagggU...................................  2     1  Mm1
.........................................................ggacggagaaGugauaagggc...................................  2     1  Mm1
.........................................................ggacggagCacugauaagggc...................................  1     1  Mm1
.........................................................ggacggagaacugauaagggA...................................  7     1  Mm1
.........................................................Ugacggagaacugauaagggc...................................  4     1  Mm1
.........................................................ggaAggagaacugauaagggc...................................  1     1  Mm1
.........................................................ggacggagaaAugauaagggc...................................  1     1  Mm1
```

```
                    Star                                 Mature
gaauuuguccgaaagcggguccucgucauuuuuccgggccuguuuuuauacccuacuggacggagaacugauaagggcucgaauaggaccaagcaacauuugacguuaucugu
.........................................................ggacggagaacugauaagggc...................................    178    0    Mm1
.........................................................ggacggagaacGgauaagggc...................................    1    1    Mm1
.........................................................ggacggagaacugauaagggcu..................................    8    0    Mm1
............................................................Uggagaacugauaagggc...................................    1    1    Mm1
```
